# The accuracy of pulse oximetry in measuring oxygen saturation by levels of skin pigmentation: a systematic review and meta-analysis

**DOI:** 10.1186/s12916-022-02452-8

**Published:** 2022-08-16

**Authors:** Chunhu Shi, Mark Goodall, Jo Dumville, James Hill, Gill Norman, Oliver Hamer, Andrew Clegg, Caroline Leigh Watkins, George Georgiou, Alexander Hodkinson, Catherine Elizabeth Lightbody, Paul Dark, Nicky Cullum

**Affiliations:** 1 School of Health Sciences, Faculty of Biology, Medicine and Health, Manchester Academic Health Science Centre, University of Manchester, Jean McFarlane Building, Oxford Rd, Manchester, M13 9PL UK; 2 NIHR Applied Research Collaboration Greater Manchester (ARC-GM), Manchester, UK; 3 Institute of Population Health, University of Liverpool, Liverpool, L69 3GF UK; 4 NIHR Applied Research Collaboration North West Coast (ARC-NWC), Manchester, UK; 5 Applied Health Research Hub, University of Central Lancashire, Preston, UK; 6 NIHR School for Primary Care Research, Division of Population Health, Health Services Research and Primary Care, School of Health Sciences, Faculty of Biology, Medicine and Health, Manchester Academic Health Science Centre, University of Manchester, Manchester, M13 9PL UK; 7 NIHR Greater Manchester Patient Safety Translational Research Centre, Division of Population Health, Health Services Research & Primary Care, University of Manchester, Manchester, M13 9PL UK; 8 Faculty of Health, University of Central Lancashire, Preston, PR1 2HE UK; 9 NIHR Manchester Biomedical Research Centre, University of Manchester, Manchester, M13 9WL UK; 10 Northern Care Alliance NHS Foundation Trust, Salford Care Organisation, Salford, M6 8HD Greater Manchester UK

**Keywords:** Pulse oximetry, Arterial blood oxygen saturation, Measurement bias, Skin pigmentation, Ethnicity, Systematic review

## Abstract

**Background:**

During the COVID-19 pandemic, there have been concerns regarding potential bias in pulse oximetry measurements for people with high levels of skin pigmentation. We systematically reviewed the effects of skin pigmentation on the accuracy of oxygen saturation measurement by pulse oximetry (SpO_2_) compared with the gold standard SaO_2_ measured by CO-oximetry.

**Methods:**

We searched Ovid MEDLINE, Ovid Embase, EBSCO CINAHL, ClinicalTrials.gov, and WHO International Clinical Trials Registry Platform (up to December 2021) for studies with SpO_2_–SaO_2_ comparisons and measuring the impact of skin pigmentation or ethnicity on pulse oximetry accuracy. We performed meta-analyses for mean bias (the primary outcome in this review) and its standard deviations (SDs) across studies included for each subgroup of skin pigmentation and ethnicity and used these pooled mean biases and SDs to calculate accuracy root-mean-square (*A*_*rms*_) and 95% limits of agreement. The review was registered with the Open Science Framework (https://osf.io/gm7ty).

**Results:**

We included 32 studies (6505 participants): 15 measured skin pigmentation and 22 referred to ethnicity. Compared with standard SaO_2_ measurement, pulse oximetry probably overestimates oxygen saturation in people with the high level of skin pigmentation (pooled mean bias 1.11%; 95% confidence interval 0.29 to 1.93%) and people described as Black/African American (1.52%; 0.95 to 2.09%) (moderate- and low-certainty evidence). The bias of pulse oximetry measurements for people with other levels of skin pigmentation or those from other ethnic groups is either more uncertain or suggests no overestimation. Whilst the extent of mean bias is small or negligible for all subgroups evaluated, the associated imprecision is unacceptably large (pooled SDs > 1%). When the extent of measurement bias and precision is considered jointly, pulse oximetry measurements for all the subgroups appear acceptably accurate (with *A*_*rms*_ < 4%).

**Conclusions:**

Pulse oximetry may overestimate oxygen saturation in people with high levels of skin pigmentation and people whose ethnicity is reported as Black/African American, compared with SaO_2_. The extent of overestimation may be small in hospital settings but unknown in community settings.

**Review protocol registration:**

https://osf.io/gm7ty

**Supplementary Information:**

The online version contains supplementary material available at 10.1186/s12916-022-02452-8.

## Background

Blood oxygen saturation levels require monitoring for health reasons in a wide range of circumstances. Low blood oxygen saturation, if identified to be hypoxemia, requires medical intervention and has been linked to an increased risk of death [1]. The gold standard measure of blood oxygen saturation levels (SaO_2_) requires a sample of arterial blood and measurement using CO-oximetry. Pulse oximetry, measuring SpO_2_ as a proxy for SaO_2_ using a non-invasive and simple device, is frequently used to detect low blood oxygen levels. Pulse oximetry has been widely used during the COVID-19 pandemic, including in non-clinical settings, to detect hypoxemia and inform decisions to escalate care [2].

The current WHO COVID-19 management guideline recommends the ‘use of pulse oximetry monitoring at home as part of a package of care’ for symptomatic people with COVID-19 [3]. Many countries have specific guidance or services for home pulse oximetry in line with this recommendation [2, 4], such as the NHS England COVID Oximetry@home service [2]. The reporting of possible bias in pulse oximetry measurement, including due to skin pigmentation, raised a growing concern about the accuracy of oxygen self-monitoring [5]. Pulse oximetry works by beaming light through skin into the blood and inferring an SpO_2_ reading from the amount of light absorbed. Higher levels of skin pigmentation could, in theory, affect how light is absorbed, thus possibly affecting the accuracy of pulse oximetry readings. Measurement inaccuracy could have serious clinical implications including the delay of urgent medical care [6]. A recent US study analysed retrospective cohort data from more than 10,000 people, comparing where a diagnosis of *occult hypoxemia* (an SaO_2_ of less than 88%) was missed by pulse oximetry [7]. Results showed people described as Black had ‘nearly three times the frequency of occult hypoxemia that was not detected by pulse oximetry’ as those described as White [7]. In November 2021, the UK Health Secretary ordered a review into racial bias in medical equipment, including pulse oximeters.

It is an important time to consider the current evidence base for the impact of skin pigmentation on the accuracy of pulse oximetry compared with the gold standard measure of SaO_2_. The only current relevant systematic review, published in 1995, included three studies that explicitly considered the impact of skin pigmentation on pulse oximetry accuracy [8]. The review suggested that pulse oximeters may overestimate blood oxygen saturation in people with dark skin [8]. The recent rapid review by the NHS Race and Health Observatory came to similar conclusions but used a non-systematic review process, i.e., no comprehensive search, risk of bias assessment or meta-analysis [6]. Our objective was to conduct a rigorous systematic review of research on the influence of skin pigmentation on the accuracy of oxygen saturation measurement by pulse oximetry (SpO_2_) compared with SaO_2_ measured by standard CO-oximetry.

## Methods

### Search strategy and selection criteria

We report this review in accordance with the Preferred Reporting Items for Systematic Reviews and Meta-Analyses (PRISMA) statement [9]. The methods used were described in the registered protocol (https://osf.io/gm7ty).

We included any methods-comparison study that compared SpO_2_ values in any population, in any care setting, measured using any type of commercially available pulse oximeter, with SaO_2_ measured by standard CO-oximetry [10]; and investigated the accuracy of pulse oximetry based on both the level of skin pigmentation and ethnic group (Additional file 1: Table S1).

We excluded studies that used (1) prototype pulse oximetry devices, (2) pulse oximeters that require high-skilled specialists to operate (such as intra-partum pulse oximetry devices), and (3) pulse oximeters used for measuring venous blood oxygen saturation. We also excluded studies that reported diagnostic test accuracy measures and those with ineligible comparators, including reference pulse oximetry, use of ineligible reference values of oxygen saturation, e.g. arterial oxygen pressure (PaO_2_), calculated SaO_2_, fractional saturation (%O_2_Hb or FO_2_Hb) [10, 11].

Following the British Standards Institution 2019 standards for pulse oximetry [10], we included data on the overall accuracy (accuracy root-mean-square, *A*_*rms*_), mean bias, precision (standard deviation of mean bias, SD) and/or the limits of agreement for the SpO_2_ and SaO_2_ comparison, with mean bias as the review’s primary outcome (Additional file 1: Table S1). The *A*_*rms*_ combines mean bias and precision in a single measure [10]. *A*_*rms*_, though being given a primacy in relation to other outcomes in the British Standards Institution standards, has no intuitive relevance to clinical decision-making. For example, an *A*_*rms*_ value of 4% means that about 68% of pulse oximetry readings would be within ± 4% of the gold standard CO-oximetry reading. To aid clinical relevance and interpretation, we use mean bias as the review’s primary outcome. The mean difference between ‘true’ blood oxygen saturation levels and pulse oximetry readings can more clearly indicate how clinical decisions referring to threshold values (e.g. admission to hospital with a pulse oximetry reading of 92% or lower) could be impacted by bias.

We identified English language reports of relevant studies through searching (1) Ovid MEDLINE, Ovid Embase and EBSCO CINAHL Plus between the inception of databases and 5 August 2021, updated to 14 December 2021, using the same search strategies (Additional file 2: Box S1); (2) the ClinicalTrials.gov and World Health Organization International Clinical Trials Registry Platform for ongoing studies in August 2021; and (3) the reference lists of retrieved included studies, relevant systematic reviews, and guideline reports. We also contacted authors of key abstracts to request further information about their studies.

Two reviewers (CS and MG, or JH, OH) independently assessed titles and abstracts of the search results for relevance and the full texts of all potentially eligible studies for inclusion, with disagreements resolved through discussion or involving a third reviewer (GN) where necessary.

### Data analysis

One reviewer (CS, or OH or JH) independently extracted data from included studies for items in Additional file 3: Box S2 and assessed the risk of bias for the included studies using an adapted QUADAS-2 (Additional file 4: Box S3) [12], all checked by another reviewer (JH, MG, OH, GN). We resolved any disagreements through discussion. Where necessary, we contacted study authors to clarify methods and data, and transformed data into a format needed for analyses, e.g. from reported 95% limits of agreement to standard deviation (SD) [13].

We pre-specified separate analysis of studies reporting level of skin pigmentation and ethnicity. When pooling data for mean bias and its SD across studies, we used the correlated hierarchical effects model with small-sample corrections under the robust variance estimation (RVE) framework. The approach enabled us to include single-measure design study data, together with multiple dependent effect size estimates of a repeated-measures design study in meta-analysis even when the dependence structure is unknown [14, 15]. We used Tau^2^, *I*^2^, the *Q* statistic and the related *χ*^2^ test to fully assess heterogeneity in meta-analysis. There is no established approach to pooling data for *A*_*rms*_ and 95% limits of agreement across studies directly. We used the pooled mean bias and the pooled SDs produced by related meta-analyses and followed the British Standards Institution methods to calculate the *A*_*rms*_[10] and Bland and Altman’s methods to calculate the population 95% limits of agreement [16]. Using R (version 4.1.2), we performed RVE meta-analyses and produced forest plots as described in Additional file 5: Box S4. When meta-analysis was not appropriate, we synthesised relevant evidence following the Synthesis Without Meta-analysis in systematic reviews (SWiM) guidance [17].

One reviewer (CS) assessed the certainty of evidence on mean bias using the GRADE approach developed for the test accuracy topic, checked by another reviewer (GN) [18, 19]. Using this approach the certainty of mean bias findings could be assessed as at high, moderate, low or very low certainty. In interpreting review findings, we used the British Standards Institution-recommended thresholds described in the Additional file 1: Table S1 to judge the accuracy of pulse oximetry [10]. With the mean bias as the primary outcome, any pooled mean bias of > 0% would indicate overestimation with pulse oximetry and a risk of missing the detection of hypoxemia whilst a mean bias of < 0% (indicating underestimation) risks over-treatment. Given pulse oximeter devices commonly present integers in percentage, we rounded pooled estimates to be integers when interpreting the related findings such as rounding mean bias values within ± 0.50 to 0%.

We analysed data on pulse oximeters of different brands/manufacturers separately where possible. We undertook pre-planned sensitivity analyses through (1) excluding studies where all participants had similar skin pigmentation or the same ethnicity, (2) excluding studies with no data available for meta-analysis without transformation, and (3) excluding studies at high overall risk of bias. We undertook post hoc sensitivity analysis by excluding studies that used descriptors of ethnicity to indicate levels of skin pigmentation. We assessed publication bias following a qualitative approach given funnel plots or Egger’s tests were not considered appropriate for this review [20].

## Results

### Study selection and characteristics

**Fig. 1:**
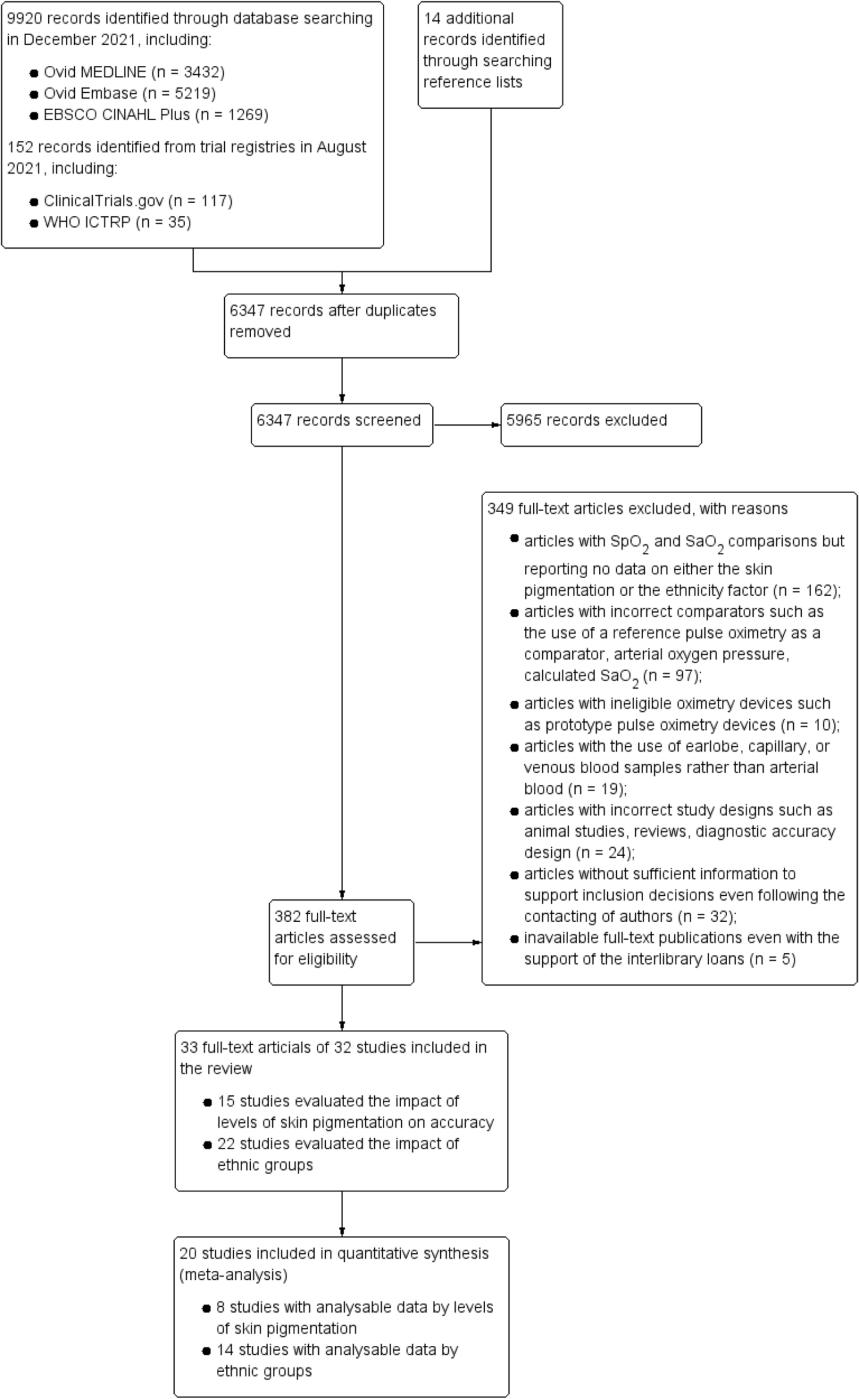
The study selection flowchart. This flowchart shows the number of records and studies at each stage of the study selection process

We assessed titles and abstracts of 9920 records identified from electronic databases, 152 from trial registries, and 14 records identified by screening the reference lists of relevant publications. Of these records, we identified 33 publications of 32 studies—published between 1985 and 2021—as eligible for inclusion (Fig. 1) [21–53]. We identified one ongoing study from electronic searches [54]. We received raw or study-level summary data for two studies directly from study authors [29, 41].

Table 1 summarises included studies, with more details in the Additional file 6: Table S2. The 32 studies (6505 participants) reported SpO_2_-SaO_2_ comparison evaluations of 54 different pulse oximeters (26 manufacturers) cf. standard SaO_2_ (Additional file 7: Table S3). Of the 32 studies, 16 (50%) reported the ranges of SaO_2_ over which the accuracy of pulse oximeters was evaluated: the minimum values of these ranges had a median of 76% whilst the maximum values had a median of 100%. Of the 16 studies, four had SaO_2_ ranges that were in line with the recommended range of 70 to 100%; eight had narrower ranges such as 80 or 90 to 100%; and four had wider ranges such as 50 or 60 to 100%.

**Table 1:** Summary characteristics of the included studies

Items	Summary statistics (*n* (%))
Study designs (32 studies)	
*Prospective design*	29 (90.62%)
*Retrospective design*	3 (9.38%)
Repeated-measures design (32 studies)	
*Yes (that is, more than one SpO*_*2*_*-SaO*_*2*_* data pair collected per person)*	21 (65.62%)
*No*	11 (34.38%)
Care settings (32 studies)	
*Hospital care settings*	27 (84.38%)
*Laboratory setting (only recruiting healthy volunteers)*	5 (15.62%)
Types of participants (32 studies)	
*Children*	7 studies (21.88%), with 1608 participants involving: • A current critical illness [23, 26, 51] • Hypoxemic conditions and/or cyanotic congenital heart disease [30, 32, 33, 45]
*Adults*	25 (78.12%), with 4897 participants involving a variety of health conditions: • Healthy volunteers [24, 29, 47, 49, 53] • Critical illnesses or conditions needing intensive care unit admission and/or mechanical ventilation [25, 27, 34–38, 46] • Pulmonary/respiratory conditions including COVID-19 [28, 39, 43, 44, 50, 52] • Cirrhosis [21], • Chronic rheumatic heart disease [48] • Postoperative hypothermia [31] • Hospitalised patients in general [22, 41] • Adults under the need of a long-term home oxygen therapy [40]
Sample sizes (32 studies)	Median 50 (range: 6 to 1562)
Age (32 studies)	
Mean or median specified (23 studies)	Median 56.40 years (range: 4 days to 69 years)
Range of arterial blood oxygen saturation SaO_2_ (%)	
SaO_2_ range specified (16 studies)	• The minimum values of the reported ranges, ranging from 50 to 94% (median 76%) • The maximum values of the reported ranges, ranging from 92 to 100% (median 100%)
Factors related to skin pigmentation (32 studies)	
*Levels of skin pigmentation*	15 studies (46.88%), with 1800 participants
*Descriptors of ethnicity*	22 studies (68.88%), with 4910 participants

### Assessment results of risk of bias and applicability

**Fig. 2:**
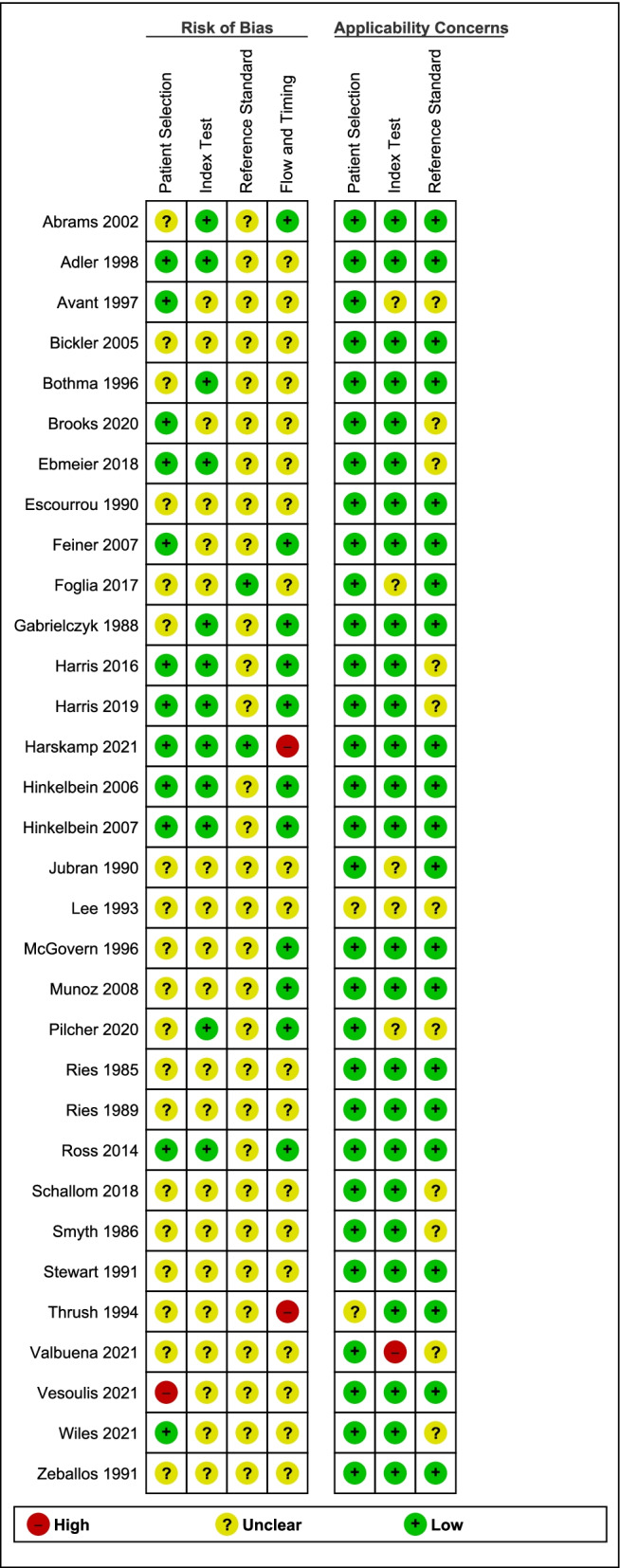
Risk of bias assessment results. The left section of this figure shows risk of bias judgements for each domain of the QUADAS-2 tool for each study and the right section shows applicability judgements for each concern domain of the QUADAS-2 tool for each study. Please see Additional file 4: Box S3 for all signalling questions used in the QUADAS-2 assessment and further considerations

Using QUADAS-2, we considered 14/32 studies (43.75%) to be at unclear risk of bias for all four domains or high risk of bias for at least one domain, and the remaining 18 (56.25%) to be at low risk of bias for at least one of the four domains (Fig. 2).

Key issues that led to downgrading for risk of bias were as follows: (1) for the patient selection domain, where specific sub-populations were inappropriately excluded from a study, or where selection criteria were unclear or not stated (19 studies); (2) for index test and reference standard domains, where there was no blinding information for either pulse oximetry SpO2 measurements (20 studies) or CO-oximeter SaO2 readings (30 studies); and (3) for the flow and timing domain, where the time intervals between SpO2 readings and the arterial blood sampling for SaO2 measurement were too long or participants were excluded from the analysis without rationale (2 studies).

We judged the applicability concern as high for one study, moderate for 13 studies, and low in terms of all three applicability considerations for the remaining 18 studies. Applicability concerns largely resulted from the lack of detail about the pulse oximeters being evaluated, CO-oximeter devices used, and/or arterial blood sampling procedures, meaning the study would be hard to reproduce.

### Pulse oximetry accuracy by levels of skin pigmentation

Fifteen of the 32 studies (1800 participants) reported by level of skin pigmentation [22, 24, 25, 27–34, 41–44, 53]. Eight of these studies (1297 participants) had available data and were included in the meta-analyses: [22, 24, 25, 27, 29, 30, 41, 42, 53] Additional file 8: Table S4 presents the mapping of originally reported terms of skin pigmentation into ‘low’, ‘medium’ or ‘high’ pigmentation categories. The remaining seven studies (503 participants) were excluded from meta-analysis due to lack of mean bias data by levels of skin pigmentation (Additional file 9: Table S5). Table 2 presents pooled accuracy data. Further details and GRADE assessment results are in Additional files 10, 11, 12 and 13: Figures S1-S3 and Table S6.

**Table 2:** Result summaries of meta-analysis for levels of skin pigmentation and ethnic groups

Subgroup categories	No. of studies (evaluations)	Sample size (data pairs)	Calculated *A*_*rms*_, %	Pooled mean bias (95% CI), %	Pooled SD (95% CI), %	Calculated 95% limits of agreement, %	Overall *I*^2^ (between-studies and within-study heterogeneity) in mean bias data pooling
**High (dark) skin pigmentation**							
Primary analysis	8 (24)	221 (3270)	1.88	1.11 (0.29 to 1.93)	1.52 (1.30 to 1.79)	− 1.87 to 4.09	98.03% (0% and 98.03%)
Sensitivity analysis, excluding studies with high overall risk of bias	6 (15)	177 (2691)	1.75	0.87 (− 0.46 to 2.19)	1.52 (1.20 to 1.93)	− 2.11 to 3.84	–
Sensitivity analysis, excluding studies with no use of standardised scales for measuring skin pigmentation	5 (9)	160 (474)	1.79	0.89 (− 1.37 to 3.14)	1.55 (1.12 to 2.15)	− 2.16 to 3.93	–
Sensitivity analysis, excluding studies with participants of high skin pigmentation alone	6 (14)	88 (2738)	1.95	1.12 (− 0.16 to 2.39)	1.60 (1.26 to 2.03)	− 2.02 to 4.26	–
**Medium pigmentation**							
Primary analysis	4 (10)	406 (1323)	1.58	− 0.58 (− 2.25 to 1.09)	1.47 (1.08 to 2.00)	− 3.46 to 2.30	92.65% (82.39% and 10.25%)
Sensitivity analysis, excluding studies with no use of standardised scales for measuring skin pigmentation	3 (4)	399 (399)	1.66	− 0.79 (− 3.03 to 1.45)	1.46 (1.00 to 2.14)	− 3.66 to 2.08	–
**Low (light) skin pigmentation**							
Primary analysis	6 (15)	670 (2865)	1.53	− 0.35 (− 1.36 to 0.67)	1.49 (1.23 to 1.81)	− 3.27 to 2.58	92.73% (22.42% and 70.31%)
Sensitivity analysis, excluding studies with high overall risk of bias	5 (12)	660 (2245)	1.57	− 0.47 (− 1.77 to 0.83)	1.50 (1.21 to 1.87)	− 3.42 to 2.47	–
Sensitivity analysis (scale)	4 (6)	648 (667)	1.62	− 0.54 (− 2.52 to 1.43)	1.53 (1.18 to 1.98)	− 3.53 to 2.45	–
**Participants described as Black/African American**							
Primary analysis	9 (22)	459 (5753)	2.27	1.52 (0.95 to 2.09)	1.68 (1.32 to 2.14)	− 1.78 to 4.82	96.39% (0% and 96.39%)
Sensitivity analysis, excluding studies with high overall risk of bias	4 (10)	67 (2892)	1.99	1.47 (− 0.21 to 3.16)	1.35 (1.08 to 1.69)	− 1.17 to 4.11	–
Sensitivity analysis, excluding studies with data transformation	7 (20)	316 (3110)	2.26	1.55 (0.85 to 2.25)	1.64 (1.28 to 2.11)	− 1.67 to 4.77	–
Sensitivity analysis, excluding studies with Black/African American populations alone	8 (16)	426 (5621)	2.35	1.63 (0.77 to 2.49)	1.69 (1.25 to 2.28)	− 1.68 to 4.94	–
**Participants from other ethnic groups, described as Asian, Hispanic or mixed ethnicity**							
Primary analysis	3 (9)	522 (2646)	1.58	0.31 (0.09 to 0.54)	1.55 (0.53 to 4.53)	− 2.72 to 3.35	47.95% (0% and 47.95%)
Sensitivity analysis, excluding studies with high overall risk of bias	2 (7)	41 (2165)	1.23	0.36 (− 0.24 to 0.95)	1.18 (0.14 to 9.78)	− 1.95 to 2.66	–
Sensitivity analysis, excluding studies with data transformation	2 (8)	488 (1405)	2.07	0.30 (− 0.80 to 1.40)	2.04 (0.32 to 12.81)	− 3.70 to 4.31	–
**White/Caucasian**							
Primary analysis	13 (48)	2195 (12,870)	1.64	0.55 (− 0.21 to 1.31)	1.55 (1.31 to 1.82)	− 2.48 to 3.58	94.39% (69.92% and 24.47%)
Sensitivity analysis, excluding studies with high overall risk of bias	8 (26)	1293 (7770)	1.42	0.36 (− 0.88 to 1.61)	1.38 (1.19 to 1.59)	− 2.33 to 3.06	–
Sensitivity analysis, excluding studies with data transformation	10 (45)	1044 (5485)	1.67	0.63 (− 0.38 to 1.63)	1.54 (1.32 to 1.80)	− 2.40 to 3.65	–
Sensitivity analysis, excluding studies with White/Caucasian populations alone	8 (16)	1246 (9791)	1.84	0.98 (− 0.08 to 2.05)	1.56 (1.16 to 2.10)	− 2.07 to 4.04	–

Hospital-based pulse oximetry probably overestimates oxygen saturation for people with high levels of skin pigmentation compared with standard SaO_2_ (8 studies, 24 comparisons, 3270 SpO_2_-SaO_2_ pairs from 221 participants): pooled mean bias 1.11% (95% CI 0.29 to 1.93%), moderate-certainty evidence. This means that, on average, pulse oximetry probably overestimates blood oxygen saturation by approximately 1%, but overestimation may be as low as 0.29% or as high as 2%. The evidence for people with medium skin pigmentation is uncertain (very low certainty evidence). The evidence for people with low levels of skin pigmentation does not suggest clinically important systematic bias (pooled mean bias -0.35, 95% CI − 1.36 to 0.67), but the finding is of low certainty. For all the levels of skin pigmentation, the *A*_*rms*_ values are around 2% or lower (95% CI non-estimable), and the pooled SD values are around 1.50% on average (Table 2). This means that, for people with any level of skin pigmentation, about 68% of their pulse oximetry readings would be within ± 2% of the CO-oximetry readings, with one SD indicating a variation around the mean bias of minus 1.50 to plus 1.50%. We tested the sensitivity of the findings: *A*_*rms*_ and SD values were generally consistent but there was increased uncertainty for mean bias findings. Additional file 14: Figure S4 presents evidence for different types of pulse oximeter: overall, most devices slightly overestimated oxygen saturation in people with high levels of skin pigmentation, with imprecision around estimates.

### Pulse oximetry accuracy by ethnicity

Twenty-two of the 32 studies (4910 participants) described participants by ethnicity rather than level of skin pigmentation [21, 23, 24, 26, 28, 29, 31, 35–40, 45–53]. We included 14 studies (3510 participants) in meta-analyses [21, 23, 24, 29, 35–37, 39, 40, 49–53]; the remaining eight (1400 participants) did not contribute to meta-analysis (Additional file 15: Table S7). Pooled data are shown in Table 2 (further data are reported in Additional files 16, 17 and 18: Figures S5-S7, and Additional file 13: Table S6). Oxygen saturation measured for people described in study reports as Black or African American may be overestimated using hospital pulse oximetry compared with standard SaO_2_ readings: mean bias 1.52% (95% CI 0.95 to 2.09%), low-certainty evidence. The 95% confidence interval of this estimate ranges between an overestimation of 1 and 2%. The evidence for people described in studies as Asian, Hispanic or of mixed ethnicity does not indicate a clinically important systematic bias (mean bias 0.31%, 0.09 to 0.54%), but it is of low certainty. The evidence is uncertain for groups described in papers as White/Caucasian, meaning further research is likely to alter findings (very low certainty evidence). The *A*_*rms*_ values are around 2% or lower (95% CI non-estimable) for all these subgroups, and the pooled SD values are around 1.50% on average (Table 2). We tested the sensitivity of the findings: *A*_*rms*_ and SD values were generally consistent but there was increased uncertainty for mean bias findings. Additional file 19: Figure S8 presents evidence for each type of pulse oximeter evaluated: overall, most devices overestimated oxygen saturation in people described as Black or African American.

## Discussion

### Summary of findings

This review suggests that for people with high levels of skin pigmentation and people described in studies as Black or African American, oxygen saturation may be overestimated by pulse oximetry in hospital compared with gold standard SaO_2_. Pulse oximetry for people with other levels of skin pigmentation is less likely to be overestimated but the evidence is uncertain. These results are for clinician-measured oximetry in controlled clinical environments and do not necessarily reflect the measurement bias of home pulse oximetry by patients or carers. The low certainty for much of the data presented means that further research could overturn these conclusions. For all the subgroups of populations evaluated, whilst the degree of mean bias is small or negligible over the ranges of SaO_2_ reported (median minimum value of 76% and maximum value of 100%), pulse oximetry readings appear unacceptably imprecise (pooled SDs > the recommended criterion of 1%) [10, 55]. Nevertheless, when the extents of measurement bias and precision are considered jointly in *A*_*rms*_, pulse oximetry measurements for all the subgroups appear acceptably accurate (with *A*_*rms*_ < the internationally recommended threshold of 4% [10, 55], or even the more conservative threshold of 3% in the US FDA guidance) [56].

### Evidence in context

Our findings have several implications. Even though our estimates suggest that the internationally recommended thresholds were met in terms of measurement bias [10, 55], the relatively small amount of mean bias identified could impact on clinical decision-making at threshold values for diagnosis of hypoxaemia. Overestimation could lead to clinically important hypoxaemia remaining undetected and untreated. Underestimated SpO_2_ readings could also be harmful, resulting in unnecessary treatment with oxygen (and the risk of hyperoxaemia) and wider impacts such as delayed hospital discharge. Two recent diagnostic studies provide evidence on clinical implications resulting from the bias in pulse oximetry for blood oxygen saturation levels [7, 57]. In these studies, people described as Black had a higher risk of ‘occult hypoxemia that was not detected by pulse oximetry’ compared with those described as White [7]. This may suggest that even small amounts of mean bias, when at the margins of diagnostic thresholds, could have an impact on diagnostic accuracy. Further understanding of these impacts could be explored via evidence synthesis of diagnostic accuracy (classification) studies to assess the clinical implications of measurement bias in relation to clinical decision-making thresholds. The amount of bias identified for people from ethnic groups such as Asian, Hispanic or mixed ethnicity appears negligible, although the certainty of the evidence is low. In terms of COVID-19 management, the 2021 WHO living guidance recommends using pulse oximetry monitoring at home as part of care package for symptomatic people in community settings but does not note the potential impact of level of skin pigmentation [3]. Our findings indicate that sub-population specific recommendations would be needed for future updates.

It is interesting to note that, despite clinically important mean bias and unacceptably large imprecision identified, the calculated *A*_*rms*_ values are generally around 2% or less over the ranges of SaO_2_ reported, that is, the *A*_*rms*_ values are far below the *A*_*rms*_ threshold of 4% required by the current international and UK standards [10, 55]. The current standards did not point out evidence sources used to underpin such requirements, but the specified values of mean bias (SD for precision) (2% (± 1%)) are consistent with the outdated 1995 Jensen review results [8]. These suggested values are even larger than the average values of our estimates (1% (± 1.5%)) in people with darker skin. Given these, currently recommended thresholds may need re-evaluation, and use of the more conservative criterion of 3% applied by the US FDA guidance may have merit [56].

Findings also support calls for better calibrating algorithms used in oximeter device software to inherently address possible measurement bias. Manufacturers should ensure, and demonstrate, that their pulse oximeters are accurate for all levels of skin pigmentation. This review results offer some insights into the possible amount of bias to consider. This however may be complex, and future work could consider a more immediate approach to clinical pathways that recognise the potential impact of small overestimations in people with darker skin.

The evidence identified has limitations in its completeness and applicability. Firstly, pulse oximetry is widely used in clinical practice and promoted for home use during the COVID-19 pandemic [2]. Many factors could theoretically affect pulse oximetry accuracy in the real world such as types of pulse oximeter probe, comorbidities, movement, age of the patient and the range of SaO_2_ levels [8]. However, most included studies in this review were based in hospital settings and had limited information whether the pulse oximeters evaluated were appropriate for home self-monitoring. This review only addresses skin pigmentation and ethnicity. Therefore, little is known for the case of pulse oximetry undertaken by untrained people at home where other factors such as movement need to be considered. Secondly, pulse oximeters have been developed and upgraded since 1970s. The included studies were published between 1985 and 2021 and some of the older studies may have used discontinued devices. Nevertheless, the overestimation of oxygen saturation for darker skin appears consistent in general across most devices evaluated. To keep the completeness of evidence in this review, we included study data for all pulse oximeter devices included.

### Strengths and limitations of this review

Before this review, our scoping exercise using a simple search of Ovid Medline with ‘pulse oximetry’ terms identified one systematic review in this area published by Jensen and colleagues in 1995 [8]. It evaluated the overall accuracy of pulse oximetry and explored possible factors that affected the accuracy. It included only one study with data on the impact of skin pigmentation, and findings were inconclusive. The comparators used for pulse oximetry measures in the Jensen review are reference measures of SaO_2_ such as PaO_2_, calculated SaO_2_ and %O_2_Hb that are now considered incorrect or outdated. We also identified a recent rapid review by the NHS Race and Health Observatory that had an unclear methodology [6]. In this rapid review, a summary of narrative findings suggested the overestimation of blood oxygen saturation levels in people with darker skin. Of the nine studies identified in this rapid review, seven had appropriate SpO_2_–SaO_2_ comparison data but the other two used inappropriate designs for the question being addressed.

Following prespecified methods to minimise the risk of bias in the review process, this review has important strengths. Our search for research is comprehensive and identified more studies. We used the gold standard CO-oximetry as the comparator for pulse oximetry, and accuracy outcomes as recommended in the British Standards Institution standards for pulse oximetry. We developed a correlated hierarchical effects model and used the novel RVE approaches to meta-analyse not only independent data (of 11 studies) but also data from studies (*n* = 21) with repeated-measures design [15]. This approach deals with correlations of multiple effect size estimates within a repeated-measures design study [14, 15].

This review has some limitations. Firstly, some included studies compared SpO_2_-SaO_2_ bias data between different subgroups of skin pigmentation or ethnicity and presented only tests of significance results, rather than SpO_2_ and SaO_2_ data per se at each subgroup level. At least two studies used diagnostic accuracy design and only presented proportions of participants with specific ranges of SpO_2_ in relation to specific SaO_2_ values, again rather than SpO_2_ and SaO_2_ data [7, 57]. We contacted authors of these studies to request relevant data and received data for two studies [29, 41]. If more data were received, then the review results could change.

Secondly, we are aware of the difference between the concepts of race and ethnicity. For simplicity, we chose to use the term of ‘ethnicity’ throughout this review given race and ethnicity are context/country-specific concepts and there is no globally accepted classification approach to distinguishing them [58]. If we had treated race and ethnicity data separately, the evidence base would change; however, we would not expect the overall conclusion to change. We also acknowledge the limitation of using scales like the Fitzpatrick scale to measure levels of skin pigmentation [59]. Such scales are criticised as being too blunt—an issue that impacts on the findings of this review and should be considered in future research.

Thirdly, we did not consider the differences between specific pulse oximeter devices, the differences between children and adults and their health conditions or the difference between skin pigmentation measurement methods. Regarding pulse oximeters evaluated, there may be differences between devices for the use of health professionals in hospitals and those for home self-monitoring. Because of these, meta-analyses in this review demonstrated between-studies heterogeneity (Table 2). However, we found, across devices evaluated and types of participants, included studies were largely consistent in suggesting oxygen saturation overestimation of using pulse oximetry. We therefore chose to pool study data, without undertaking further subgroups for these differences.

Fourthly, we only searched for English language peer-reviewed publications, without considering preprints. However, there are probably no major differences between summary treatment effects in English-language restricted meta-analyses and other language-inclusive meta-analyses [60], and the exclusion of non-English language publications from systematic reviews had no impacts on overall findings [61]. We considered the possible publication bias in assessing the certainty of evidence using GRADE approach.

Finally, no available approach to risk of bias and GRADE assessment is specific to the topic of this review. We were only able to use the relevant approaches developed for the test accuracy topic, and the GRADE approach used was only applicable to assess the certainty of evidence for mean bias, rather than precision, *A*_*rms*_ and limits of agreement.

## Conclusions

Pulse oximetry may overestimate blood oxygen saturation levels for people with dark skin in hospital settings compared with gold standard SaO_2_ measures. The evidence for the measurement bias identified for other levels of skin pigmentation or ethnicities is more uncertain. Whilst the extent of measurement bias and overall accuracy meet current international thresholds, the variation of pulse oximetry measurements appears unacceptably wide. Such a small overestimation may be crucial for some patients: particularly at the threshold that informs clinical decision-making.

## Supplementary Information

**Additional file 1: Table S1.** Measure, definition, and data formats to assess the accuracy of pulse oximetry compared with reference measures.

**Additional file 2: Box S1.** The Ovid MEDLINE search strategy.

**Additional file 3: Box S2.** Data items in the data extraction form.

**Additional file 4: Box S3.** The QUADAS-2 tool used for assessing risk of bias and applicability with further explanations.

**Additional file 5: Box S4.** Data synthesis methods and generic R codes used.

**Additional file 6: Table S2.** Characteristics of the included studies.

**Additional file 7: Table S3.** Types of pulse oximeters and CO-oximetry evaluated in the included studies.

**Additional file 8: Table S4.** Mapping terms originally used for indicating skin pigmentation into low, medium or high level of skin pigmentation defined in the review for meta-analysis.

**Additional file 9: Table S5.** Evidence from studies where skin pigmentation measures cannot be specified or grouped into low, medium, and/or high pigmentation.

**Additional file 10: Figure S1.** Summary presentations of study sample sizes (n) and numbers of data pairs compared (N), accuracy root mean square (Arms), mean bias (SD) and limits of agreement (LoA) of pulse oximeters for the subgroup of high (dark) skin pigmentation.

**Additional file 11: Figure S2.** Summary presentations of study sample sizes (n) and numbers of data pairs compared (N), accuracy root mean square (Arms), mean bias (SD) and limits of agreement (LoA) of pulse oximeters for the subgroup of medium skin pigmentation.

**Additional file 12: Figure S3.** Summary presentations of study sample sizes (n) and numbers of data pairs compared (N), accuracy root mean square (Arms), mean bias (SD) and limits of agreement (LoA) of pulse oximeters for the subgroup of low (light) skin pigmentation.

**Additional file 13: Table S6.** Summary of findings table for the impact of skin pigmentation and ethnicity on the accuracy of pulse oximetry compared with CO-oximetry.

**Additional file 14: Figure S4.** Summary presentations of study sample sizes (n) and numbers of data pairs compared (N), accuracy root mean square (Arms), mean bias (SD) and limits of agreement (LoA) of pulse oximeters for levels of skin pigmentation by the different types of pulse oximeters.

**Additional file 15: Table S7.** Evidence from studies that could not be included in quantitative data pooling for the ethnicity factor.

**Additional file 16: Figure S5.** Summary presentations of study sample sizes (n) and numbers of data pairs compared (N), accuracy root mean square (Arms), mean bias (SD) and limits of agreement (LoA) of pulse oximeters for the subgroup of Black/African American ethnic groups.

**Additional file 17: Figure S6.** Summary presentations of study sample sizes (n) and numbers of data pairs compared (N), accuracy root mean square (Arms), mean bias (SD) and limits of agreement (LoA) of pulse oximeters for the subgroup of non-Black, non-White ethnic groups.

**Additional file 18: Figure S7.** Summary presentations of study sample sizes (n) and numbers of data pairs compared (N), accuracy root mean square (Arms), mean bias (SD) and limits of agreement (LoA) of pulse oximeters for the subgroup of White/Caucasian ethnic groups.

**Additional file 19: Figure S8.** Summary presentations of study sample sizes (n) and numbers of data pairs compared (N), accuracy root mean square (Arms), mean bias (SD) and limits of agreement (LoA) of pulse oximeters for ethnic groups by the different types of pulse oximeters.

## Data Availability

All relevant data are within the manuscript and its Additional files. No additional data available.

## Abbreviations

*A*_*rms*_: Accuracy root-mean-square
FDA: Food and Drug Administration
%O_2_Hb or FO_2_Hb: Fractional saturation
PaO_2_: Arterial oxygen pressure
PRISMA: Preferred Reporting Items for Systematic Reviews and Meta-Analyses
RVE: Robust variance estimation
SaO_2_: Gold standard measure of blood oxygen saturation levels
SD: Standard deviation
SpO_2_: Blood oxygen saturation levels measured using pulse oximetry
SWiM: Synthesis Without Meta-analysis in systematic reviews
