# Supplementary material for: The accuracy of pulse oximetry in measuring oxygen saturation by levels of skin pigmentation: a systematic review and meta-analysis
Source: BMC Med. 2022 Aug 16;20:267. doi: 10.1186/s12916-022-02452-8 (PMC9377806; doi:10.1186/s12916-022-02452-8)
Supplement: Supplementary file 1 — Additional file 1: Table S1. Measure, definition, and data formats to assess the accuracy of pulse oximetry compared with reference measures. [file 12916_2022_2452_MOESM1_ESM.docx]

## **Table S1. Measure, definition, and data formats to assess the accuracy of pulse oximetry compared with reference measures**

| **Measure** | **Definition** | **Eligible data** |
| --- | --- | --- |
| The overall accuracy of pulse oximeter equipment | Agreement between a test result and an accepted reference value. That is a combination of a random component and of a common systematic error or bias component. | Expressed as the root-mean-square difference between measured values (SpO_2_) and reference values (SaO_2_). |
| Bias | Overestimation or underestimation of test measurement method relative to a reference measure. That is the total systematic error. | Assessed as mean difference between two measures (between-test mean, in this case SaO_2_-SpO_2_): a larger systematic difference means a larger bias value. |
| Precision | Closeness of agreement between independent test results obtained, which reflects the variability between test measures. Precision depends on random errors. | Reported as the standard deviation of between-test mean difference and a larger standard deviation means less precision. |
| Agreement | Concordance between two sets of measurements | Expressed as the limits of agreement (via the use of Bland-Altman plots) between different measures |

**Notes on considerations and criteria used in defining skin pigmentation and ethnicity and in choosing accuracy outcomes**

In this review, we focus on reporting the comparative accuracy of pulse oximetry-produced SpO_2_ cf. standard SaO_2_ by levels of skin pigmentation and ethnic groups. We considered ethnicity as a sub-optimal, but relevant, proxy for level of skin pigmentation in relation to the accuracy of the biological reading of oxygen saturation.

For level of skin pigmentation measurement, we included studies that used a standardised measure of level of skin pigmentation such as the Fitzpatrick scale,[12] and studies that used an unstandardised or qualitative judgement of skin pigmentation levels such as so called ‘light’ or ‘dark’ level of skin pigmentation. The originally reported terms of skin pigmentation were mapped into ‘low’, ‘medium’ or ‘high’ pigmentation categories, without any further re-classification. For example, if a study classified skin as ‘light’ and ‘dark’, then the light skin group was mapped into the low pigmentation category, and the dark skin group into the high pigmentation category.

For ethnicity, we included studies that grouped participants based on any ethnicity classification. We interpreted terms such as Black and White as describing ethnicity and only indirect indicators of skin pigmentation.[13]

We prespecified accuracy outcomes evaluated following the British Standards Institution 2019 standards for pulse oximetry.[10] The outcomes chosen were defined in Table S1. They include:

- the overall accuracy, agreement between a test result and an accepted reference value reported as accuracy root-mean-square, *A_rms_*, which is a combination of mean bias and precision in a single measure.[10]
- mean bias, calculated as the mean difference between two measures (in this case SpO_2_ – SaO_2_).[10] A larger mean difference indicates a larger bias value and values greater than 0 indicate overestimation with pulse oximetry.[10]
- precision, commonly reported as the standard deviation (SD) of between-test mean difference with a larger standard deviation indicating less precision.[10]
- the limits of agreement for the SpO_2_ and SaO_2_ comparison.

For pulse oximetry to be accurate by the British Standards Institution-recommended thresholds, the overall accuracy *A_rms_* should be within 4% over the range of 70% to 100% SaO_2_. For the mean bias (and precision), SpO_2_ measures should be within +/- 2% of CO-oximetry measures and the variation for repeated SpO_2_ measures should be within one SD of the mean bias.[10]

The British Standards Institution standards for pulse oximetry gives *A_rms_* primacy, as it details general accuracy and thus the suitability of the machine for its purpose. The value is a root mean square deviation calculation, and the relevance of this measure to clinical decision-making is not intuitive. Because of this we present mean bias as the review’s primary outcome. This mean difference between ‘true’ blood oxygen saturation levels and those measured by pulse oximetry can more clearly indicate how clinical decisions referring to threshold values (e.g., admission to hospital with a pulse oximetry reading of 92% or lower) could be impacted by bias.
