# Supplementary material for: The accuracy of pulse oximetry in measuring oxygen saturation by levels of skin pigmentation: a systematic review and meta-analysis
Source: BMC Med. 2022 Aug 16;20:267. doi: 10.1186/s12916-022-02452-8 (PMC9377806; doi:10.1186/s12916-022-02452-8)
Supplement: Supplementary file 2 — Additional file 2: Box S1. The Ovid MEDLINE search strategy. [file 12916_2022_2452_MOESM2_ESM.docx]

## **Box S1. The Ovid MEDLINE search strategy**

| 1 exp Oximetry/  2 (oximet* or oxymet*).ti,ab,kw.  3 (SpO2 or %spo2 or sp o2).tw.  4 or/1-3  5 (co-oximet* or co-oxymet* or h?emoximet*).ti,ab,kw.  6 (blood adj3 (analys* or measure*)).tw.  7 (blood sampl* or gold standard or reference device* or reference instrument* or in-line oximet* or in vitro oximet* or arterial oxygen saturation or arterial oxyhemoglobin saturation or arterial oxyhaemoglobin saturation or arterial blood or arterial puncture or SaO2 or %SaO2 or sa o2).tw.  8 or/5-7  9 Reproducibility of Results/  10 Validation Study/  11 Evaluation Studies as Topic/  12 Bias/  13 "Sensitivity and Specificity"/  14 Hypoxia/di [Diagnosis]  15 comparative study.pt.  16 (accura* or inaccura* or overestimat* or over-estimat* or underestimat* or under-estimat* or agreement or root-mean-square or root mean square or RMS or quadratic mean).tw.  17 (precision or evaluat* or predict* or reliab* or reproducib* or concordance or performance or bias or validat* or error* or erroneous or individual variability or (variability and (analysis or values)) or sensitivity or specificity or failure).tw.  18 (compar* adj3 (measure* or value*)).tw.  19 (controlled desaturation or paired repeated measure* or method comparison or calibration stud*).ti,ab,kw.  20 (paired readings or paired measurements or "difference of values" or "limits of agreement" or "limits of values" or confidence limits or regression or bland altman).ti,ab,kw.  21 or/9-20  22 4 and 8 and 21  23 exp animals/ not humans.sh.  24 22 not 23  25 limit 24 to english language |
| --- |
