# Supplementary material for: The accuracy of pulse oximetry in measuring oxygen saturation by levels of skin pigmentation: a systematic review and meta-analysis
Source: BMC Med. 2022 Aug 16;20:267. doi: 10.1186/s12916-022-02452-8 (PMC9377806; doi:10.1186/s12916-022-02452-8)
Supplement: Supplementary file 3 — Additional file 3: Box S2. Data items in the data extraction form. [file 12916_2022_2452_MOESM3_ESM.docx]

## **Box S2. Data items in the data extraction form**

| - basic characteristics of studies, including first author, publication type, publication year, accuracy study type (lab-based, controlled desaturation study in healthy volunteers vs real-world accuracy study in patients); - study setting; - characteristics of participants, including eligibility criteria particularly health conditions, the number of participants and/or the number of pairs of oxygen saturation measures, and average age of participants; - pulse oximetry tests being compared, including oximeters used and their manufacturers and models if available, probe type (transmissive vs reflectance probe), and location of sensor (e.g. finger, ear, toe); - methods used for measuring SaO_2_ including the blood gas analyser and CO-oximeter model, and the blood source such as the radial artery; - skin pigmentation definitions including scales used and pigmentation levels, as well as, those for race/ethnicity; - data on accuracy, bias and precision of measurement at a study level including comparative data based on level of skin pigmentation and that of race/ethnicity group as reported - other outcome data available by level of skin pigmentation or by race/ethnicity groups - unit of analysis (either individuals, or repeated measurements); and - other factors that were reported to have the effects on pulse oximetry accuracy. |
| --- |
