# Supplementary material for: The accuracy of pulse oximetry in measuring oxygen saturation by levels of skin pigmentation: a systematic review and meta-analysis
Source: BMC Med. 2022 Aug 16;20:267. doi: 10.1186/s12916-022-02452-8 (PMC9377806; doi:10.1186/s12916-022-02452-8)
Supplement: Supplementary file 4 — Additional file 4: Box S3. The QUADAS-2 tool used for assessing risk of bias and applicability with further explanations. [file 12916_2022_2452_MOESM4_ESM.docx]

## **Box S3. The QUADAS-2 tool used for assessing risk of bias and applicability with further explanations**

| **Domain 1. Participant selection**: could the selection of participants have introduced bias?  Signalling questions for assessing risk of bias:   1. *Was an appropriate sample of participants included in the study?* ^a^ 2. Was a case-control design avoided? (omitted) ^b^ 3. Did the study avoid inappropriate exclusions? ^c^   Signalling questions for assessing applicability:   - Are there concerns that the included participants and settings do not match the review question?   **Domain 2. Index test**: could the conduct or interpretation of the index test have introduced bias?  Signalling questions for assessing risk of bias:   1. *Were the pulse oximetry results interpreted without knowledge of the results of the reference standard?* 2. If a threshold was used, was it pre-specified? (omitted) ^d^   Signalling questions for assessing applicability:   - Are there concerns that the index test, its conduct, or its interpretation differs from the review question?   **Domain 3. Reference standard**: could the reference standard, its conduct, or its interpretation have introduced bias?  Signalling questions for assessing risk of bias:   1. *Is the reference standard likely to correctly measure the blood oxygen saturation level?* ^e^ 2. *Were the reference standard results interpreted without knowledge of the results of pulse oximetry?*   Signalling questions for assessing applicability:   - Are there concerns that the target condition as defined by the reference standard does not match the question?   **Domain 4. Flow and timing**: could the analysis of flow and timing have introduced bias?  Signalling questions for assessing risk of bias:   1. *Was there an appropriate interval between pulse oximetry and reference standard? In this case it is considered appropriate that the index and reference standard measures are taken at the same time with no obvious time interval between them.* 2. Did all patients receive a reference standard? 3. Did all patients receive the same reference standard? 4. Were all patients included in the analysis? ^f^ |
| --- |

Notes:

a. To make the question more relevant for this accuracy review the wording has been amended. BSI for pulse oximetry allows for two types of participants for evaluating the SpO_2_ accuracy:[10] healthy volunteers in a controlled desaturation study, and patients in clinical care settings. A study needs to define, select and recruit participants of interests accordingly. A study involving patients in clinical care settings may use consecutive or random sampling.

b. We omitted this question as it is more relevant to diagnostic test accuracy (DTA) reviews than this accuracy question.

c. For the pulse oximetry accuracy review, some studies may aim to evaluate the accuracy of pulse oximetry in people with a range of characteristics (e.g. level of skin pigmentation, baseline oxygen saturation levels), but inappropriately exclude important subgroups. This inappropriate exclusion may result in biased results in pulse oximetry accuracy. Therefore, new criteria may be applied to this signalling question even though the wording is unchanged from the original QUADAS-2 tool.

d. We omitted this question as it is more relevant for DTA reviews than this accuracy review.

e. Classification is particularly relevant to DTA. Pulse oximetry is used to measure (not classifying) SpO_2_. Therefore, the question is slightly re-worded by replacing ‘classify’ with ‘measure’.

For the pulse oximetry accuracy topic it is more relevant to consider how likely pulse oximetry SpO_2_ measurement follows recommended procedures, and if applicable, is carried out under appropriately standardised conditions. For example, BSI 2019 standards states, when the oxygen saturation needs to be changed to another level, there needs to be at least 30 s to allow SaO_2_ to reach stability before the pulse oximeter reading is taken.[10] Similarly, blood sampling can begin only when the blood saturation stabilises at an acceptable level.

f. In this review we considered whether excluded data are considered ‘eligible’ for exclusion (with appropriate justifications) according to BSI for pulse oximetry guidance.[10] For example, for pulse oximeter monitors that set up an upper limit on displayed SpO_2_ (e.g. 99 %), data collected with SaO_2_ values beyond the specified SpO_2_ limit legitimately be excluded. Data pairs can be excluded if they were taken under conditions that were outside of the pre-planned test scope.
