# Supplementary material for: The accuracy of pulse oximetry in measuring oxygen saturation by levels of skin pigmentation: a systematic review and meta-analysis
Source: BMC Med. 2022 Aug 16;20:267. doi: 10.1186/s12916-022-02452-8 (PMC9377806; doi:10.1186/s12916-022-02452-8)
Supplement: Supplementary file 5 — Additional file 5: Box S4. Data synthesis methods and generic R codes used. [file 12916_2022_2452_MOESM5_ESM.docx]

## **Box S4. Data synthesis methods and generic R codes used**

| **Part 1. Data synthesis methods used**  In this review, we performed meta-analyses for mean bias of SpO_2_-SaO_2_ and their SDs across included studies and used their pooled estimates to calculate overall accuracy and 95% limits of agreement. We described specific methods used below.  ***(1) Meta-analysis for mean bias of SpO_2_-SaO_2_ and SDs***  We pooled study data of mean bias of SpO_2_-SaO_2_ for assessing whether use of pulse oximetry would under-estimate (pooled mean bias < 0) or over-estimate (pooled mean bias > 0) oxygen saturation in relation to CO-oximetry. We pooled study data on the SDs of mean bias for assessing the precision of pulse oximetry measures. For either mean bias or SD data pooling, we used the random-effects, correlated hierarchical effects model with small-sample corrections of the Robust Variance Estimation (RVE).[17] We chose this approach because   - we expected to include studies with repeated measures design, thus obtaining multiple dependent effect size estimates – data on mean bias and SD – within a study; - we did not expect to have data on correlations between multiple dependent effect size estimates within a study as such correlations are commonly not reported in included studies. Therefore, the exact dependence structure of the multiple effect sizes within a study is unknown, and the conventionally used multivariate meta-analysis with known dependence structure of effect sizes could not be used in the case of this review. - The approach we chose is a hybrid of correlated hierarchical effects model and RVE methods. Correlated hierarchical effects model allowed us to construct a flexible variance structure that could better capture two types of dependence: hierarchical effects and correlated effects. The RVE framework used modelling of dependence to approximate the unknown dependence structure and the structure does not have to be fully correct. Even if the structure is mis-specified, RVE’s regression coefficient estimates (effect size) could be unbiased and standard errors could validly quantify uncertainty.[16] RVE approach uses products of the regression residuals to roughly approximate the variance-covariance structure of the errors (i.e. producing standard errors).[17] This estimation of standard errors is separated from the choice of weight matrices and therefore, standard errors can be produced without having to know the dependence structure.   More specifically we used the following methods to analyse data:  First, we used the method described by Tipton and Shuster to adjust dependent standard deviations, by which the under-estimation bias of the true standard deviation could be reduced in the case of repeated measures design.  To obtain mean bias and its variance estimates for each study,   - mean bias = SpO_2_-SaO_2_ difference as reported - ${adjusted SD}^{2}= {reported SD}^{2}[(the total number of repeated measures-1)/(the total number of repeated measures-the number of replications per participant)]$   E.g. in a study with 10 participants and a total of 200 repeated measures for a pulse oximetry, the number of replications per participant is 20 (i.e. 200/10).   - the sampling variance of mean bias = adjusted SD^2^ / the number of participant.   In producing SD and its variance estimates for each study, we used their log-transform to normalise the distribution and stabilise its variance:   - log(adjusted SD^2^)$\approx\log\left( adjusted {SD}^{2} \right)+\frac{1}{the numbe of participants-1}$ - the sampling variance of log(adjusted SD^2^) $\approx\frac{2}{the numbe of participants-1}$   Second, we performed analyses in this section using rma.mv() function available in the package of *metafor.*  For pooling data on either mean bias or SD, we performed multi-level random-effects models without moderators for the dataset of either each level of skin pigmentation or ethnic group, where there was at least one study with at least two sets of effect size estimates (i.e. mean bias, or SD). For this, we used restricted maximum likelihood estimation in the function rma.mv().  By including a random term, multi-level random-effects model is specifically designed to deal with dependence among multiple effect size estimates within a study. We considered multiple effect size estimates within a study having random effects, thus including its indicator as a random term in each model to deal with their dependence.  For constructing reasonable multi-level random-effects models, we specified variance components with a correlation of 0.90. The correlation value resulted from the Jensen review.[8]  We used the Tau^2^ statistic, produced by multi-level random-effects models, to quantify heterogeneity.  Third, based on multi-level random-effects model outputs, we finally used RVE approaches – more specifically the package of *clubSandwich* – to estimate the RVE standard errors.  ***(2) Calculations of overall accuracy and 95% limits of agreement***  There was no acceptable meta-analysis method to produce overall accuracy and 95% limits of agreement directly from study-level data of mean bias and SD for the case of this review.  When obtaining the pooled mean bias and the pooled SD for either each level of skin pigmentation or each ethnic group, we calculated overall accuracy using the BSI recommended method:[10]   - *A_rms_* for the overall accuracy = $\sqrt{({the pooled mean bias}^{2} + {the pooled SD}^{2})}$   We also calculated the 95% limits of agreement using the following method described by Bland and Altman.[18]   - 95% limits of agreement = pooled mean bias ± 1.96 * pooled SD   **Part 2. Generic R codes (bold and *Italic* texts) used for meta-analyses**  ## Load R packages required  ***library(clubSandwich)*** ### This package was for the robust variance estimation (RVE) approach  ***library(metafor)***  ## Load corresponding data used for meta-analysis of each level of skin pigmentation and each ethnicity group, respectively  dataset  ## Run a multilevel random effects model (constant sampling correlation) for either bias or SD  ***V_mat <- impute_covariance_matrix(dataset$Vi,***  ***cluster = dataset$level1,***  ***r = 0.9,***  ***smooth_vi = TRUE)***  ***multilevel_model <- rma.mv(yi ~ 1,***  ***V = V_mat,***  ***random = ~ 1 \| level1/ level2,***  ***data = dataset, sparse = TRUE, slab=paste(level1))***  ***multilevel_model*** ### standard errors produced by these were model-based, rather than RVE ouputs  ## the estimation CIs of tau2 for between-studies heterogeneity and within-study heterogeneity  ***confint(multilevel_model)***  ## the calculation of I^2^ for an overall model  ***W <- diag(1/dataset$Vi)***  ***X <- model.matrix(multilevel_model)***  ***P <- W - W %*% X %*% solve(t(X) %*% W %*% X) %*% t(X) %*% W***  ***100 * sum(multilevel_model$sigma2) / (sum(multilevel_model$sigma2) + (multilevel_model$k- multilevel_model$p)/sum(diag(P)))***  ## the separation of the overall I2 for between-studies and within-study heterogeneity  ***100 * multilevel_model$sigma2 / (sum(multilevel_model$sigma2) + (multilevel_model$k- multilevel_model$p)/sum(diag(P)))***  ## the calculation of RVE standard errors  ***CI_multilevel_model <- conf_int(multilevel_model, vcov = "CR2")***  ***CI_multilevel_model*** |
| --- |
