# Supplementary material for: The accuracy of pulse oximetry in measuring oxygen saturation by levels of skin pigmentation: a systematic review and meta-analysis
Source: BMC Med. 2022 Aug 16;20:267. doi: 10.1186/s12916-022-02452-8 (PMC9377806; doi:10.1186/s12916-022-02452-8)
Supplement: Supplementary file 6 — Additional file 6: Table S2. Characteristics of the included studies. [file 12916_2022_2452_MOESM6_ESM.docx]

## **Table S2. Characteristics of the included studies**

| Study | Study (test) settings | Pulse oximeter models evaluated | CO-oximetry | Blood source | Participant inclusion criteria | Average age | Range of arterial blood oxygen saturation SaO_2_ (%) | Factors of interest and measurements | Other factors |
| --- | --- | --- | --- | --- | --- | --- | --- | --- | --- |
| Abrams 2002 ^23^ | NR | 1 model:  Nellcor N-200 | Radiometer ABL520 | Arterial blood | Adult patients with cirrhosis (n = 294) | Mean 51.7 years | Not reported | Race (White, Black) | Cirrhosis or not, oxygen saturation levels, hemoglobin levels, hepatopulmonary syndrome or not |
| Adler 1998 ^24^ | A & E | 1 model:  Nellcor D-25 | 4-wavelength spectro-photometer, or co-oximeter (Radiometer OSM3) | Arterial blood (no further detail) | Adult patients who needed blood gas analysis (n = 284) | Mean 60 years | 50 to 99 | Skin pigmentation measured using the Munsell colour system with categories of light, medium, or dark | NR |
| Avant 1997 ^25^ | Hospital or wards | 2 models:  Nellcor Oxiband, Nellcor Dura-Y | CO-oximeter | Arterial blood | Critically ill children (n = 50) | Mean 26 months | 80 to 100 | Race (White, Black) | NR |
| Bickler 2005 ^26^ | Lab | 5 models:  Nellcor N-595 with Nellcor OxiMax A finger probe;  Two types of Novametrix 513s models;  Two types of Nonin Onyx models | Radiometer OSM3 | Arterial blood (radial or other arteries) | Healthy, non-smoking volunteers (n = 21) | Mean 29.05 years | 60 to 100 | Race/ethnicity and skin pigmentation with categories of light (northern European) and dark (African-American) | Oxygen saturation levels (SaO_2_ 60 –70%, 70–80%,  80–90%, and 90–100%) |
| Bothma 1996 ^27^ | Hospital or wards | 3 models:  Simed S100e;  Nihon Koden;  Ohmeda 3740 | IL482 co-oximeter | Arterial blood (no further detail) | Darkly pigmented critically ill adult patients (n = 100) | Adults, age not reported | 88 to 99 | All dark pigmentation objectively quantified using EEL reflectance spectrophotometer (Evans Electroselenium Company) | NA |
| Brooks 2020 ^28^ | Hospital or wards | 2 models:  Masimo, Nellcor (Covidien) | Radiometer ABL800 co-oximeter | Arterial blood | ICU infants and children (n = 929) | Median 1.9 years | 70 to 100 | Ethnicity (Aboriginal and/or Torres Strait Islander (ATSI), not ATSI) | Health conditions, age at admission, weight at admission, sex, sensor type (Massimo, Nellcor), SaO2 category, lactate, total haemoglobin (Hb), pH, oxygen saturation index, ventilation, inotropes, vasodilators, and vasoconstrictors |
| Ebmeier 2018 ^29^ | Hospital or wards | 2 models:  Masimo oximeter for GE Marquette Rac-4A monitor;  Philips sensors for Philips IntelliVue MP70 monitor | Radiometer ABL 800 FLEX arterial blood gas analyser | Arterial blood (no further detail) | Consecutive ICU patients (n = 394) | Mean 62.5 years | 85 to 100 | Skin pigmentation measured using the Fitzpatrick scale with categories of light (score of 1 or 2), medium (score of 3 or 4), and dark (score of 5 or 6) | PaO_2_, acute physiology and chronic health evaluation (APACHE) II illness severity score, use of vasopressors, use of inotropes, capillary refill time (> vs < 3 seconds), body temperature, temperature of the hands, mean arterial pressure, pulse pressure, local factors |
| Escourrou 1990 ^30^ | Hospital or wards | 3 models:  Ohmeda Biox 3700;  Criticare CSI 501+;  Nellcor N-200 | Radiometer OSM2 | Arterial blood (radial or other arteries) | Adult patients with chronic pulmonary diseases (n = 101) | Range: 17 to 81 years | Not reported | Skin pigmentation with categories of moderate vs unclear (but not Black) level | Exercise loads |
| Feiner 2007 ^31^ | Lab | 3 models (6 types of finger probe):  Nellcor N-595 (OxiMax A adhesive probe);  Nellcor N-595 (a clip-type probe);  Masimo Radical (clip probe);  Masimo Radical (adhesive probe);  Nonin 9700 (clip-type probe);  Nonin 9700 (adhesive probe) | Radiometer OSM3 | Arterial blood (radial or other arteries) | Healthy non-smoking volunteers (n = 36) | Mean 29 years | 60 to 100 | Race/ethnicity and skin pigmentation defined as light (Caucasian), intermediate (Hispanic, Indian, Filipino, Vietnamese), and dark (African American) categories | Oxygen saturation levels, gender |
| Foglia 2017 ^32^ | Hospital or wards | 2 models:  Nellcor Oximax (Covidien);  Masimo Rainbow SET Radical 7 | Siemens Rapidlab 1265 | Arterial blood (no further detail) | Infants with cyanotic congenital heart disease and oxygen saturation <90% (n = 36) | Mean 6 days in light pigment, 118 days in dark pigment | 60 to 92 | Skin pigmentation measured using the Munsell Soil Book of Colour, Hue 7.5YR, with categories of light and dark | Oxygen saturation levels |
| Gabrielczyk 1988 ^33^ | Hospital or wards | 1 model:  Nellcor N-100 | Radiometer OSM2 | Arterial blood (radial or other arteries) | Patients with postoperative hypothermia (n = 21) | Mean 59.5 years | 90 to 100 | Skin pigmentation with categories of racially pigmented skin vs unclear pigmentation level | NR |
| Harris 2016 ^34^ | Hospital or wards | 3 models:  Masimo SET with LNCS sensor;  Masimo SET Blue sensor;  Nellcor N-600 Max-I sensor | AVOXimeter 1000E co-oximeter | Arterial blood (no further detail) | Hypoxemic pediatric patients with cyanotic congenital heart disease (n = 50) | Mean 18 months | Not reported | Skin pigmentation measured using the Massey Skin Colour Score (categorised to be four levels) | Age, height, weight, binary indicators of non-White race and female gender |
| Harris 2019 ^35^ | Hospital or wards | 2 models:  Masimo LNCS sensor;  Nonin WristOx2 3150 with Bluetooth-enabled infant sensors 8008J | Bedside co-oximetry | Arterial blood (no further detail) | Hypoxemic infants with cyanotic heart disease (n = 24) | Median 13 days | Not reported | Skin pigmentation measured using the Massey Skin Colour score (NR) | Age, sensor placement |
| Harskamp 2021 ^36^ | Hospital or wards | 11 models:  AFAC FS10D, AGPTEK FS10C, ANAPULSE ANP 100, Cocobear, Contec CMS50D1, HYLOGY MD-H37, Mommed YM101, PRCMISEMED F4PRO, PULOX PO-200, Zacurate Pro Series 500 DL, Philips M1191BL | Radiometer ABL90 Flex Plus | Arterial blood (radial or other arteries) | Intensive care patients (n = 35) | Mean 69 years | 86 to 100 | Skin pigmentation measured using the Fitzpatrick scale, with two categories: dark skin type (Fitzpatrick scale IV-VI) vs non-dark skin (Fitzpatrick I-III) | Age, sex, heart rate bias, body temperature, cold hands to touch, systolic blood pressure, and use of vasopressor drugs |
| Hinkelbein 2006 ^37^ | Hospital or wards | 2 models:  Nellcor DS-100A Durasensor sensor with SIEMENS SC1281 monitor (SIREM module); Philips M1191A finger probe (PHILIPS IntelliVue MP70 monitor) | Radiometer ABL625 | Arterial blood | ICU adults with mechanical ventilation (n = 46) | Mean 58.1 years | 91 to 99 | Race - all White (Caucasian) | NR |
| Hinkelbein 2007 ^38^ | Hospital or wards | 1 model:  Nellcor DS-100A Durasensor sensor with SIEMENS SC1281 monitor (SIREM module) | Radiometer ABL625 | Arterial blood (radial or other arteries) | ICU adults with mechanical ventilation (n = 50) | Mean 59 years | 94 to 100 | Race - all White (Caucasian) | NR |
| Jubran 1990 ^39^ | Hospital or wards | 2 models:  Nellcor pulse oximeter, Ohmeda-Biox3700 pulse oximeter | CO-oximetry | Arterial blood | Critically ill, ventilator-dependent patients (n = 54) | Mean 53 years | Not reported | Ethnicity – Black, and White categories | NR |
| Lee 1993 ^40^ | Hospital or wards | 3 models:  Nellcor, Simed, Critikon | Nova Stat Profile 3 pH/blood gas analyser | Arterial blood | ICU adults (n = 33) | Mean 56.4 years | Not reported | Race (Chinese, Indian, Malay) | Hypoxia levels |
| McGovern 1996 ^41^ | Hospital or wards | 1 model:  Ohmeda 3700 | IL 482 Co-oximeter | Arterial blood (radial or other arteries) | Adults with stable condition with severe COPD (n = 8) | Mean 63.2 years | Not reported | Race - all White | Exercise workload |
| Muñoz 2008 ^42^ | Hospital or wards | 1 model:  Minolta Pulsox-7 | IL 682 co-oximeter | Arterial blood (radial or other arteries) | Adults under assessment for long-term home oxygen therapy (n = 846) | Mean 68.4 years | Not reported | Race – all Caucasian | Arterial oxygen tension and PaCO2, methods of measuring oxygen saturation (Oximeter vs co-oximeter ) |
| Pilcher 2020 ^43^, Ploen 2016 (abstract) ^44^ | Hospital or wards | 14 models:  Carescape B450 monitor with Nellcor probe;  GE Dash 3000;  Masimo Radical 7;  Masimo SET Quartz (unspecified);  Masimo SET Quartz Q400;  Nonin 2120;  Nonin 2140;  Nonin Avant (unspecified);  Nonin Avant 4000;  Nonin Avant 9700;  Nonin Lifesense Medair;  Novametrix Model 512;  Ohmeda Biox 3700E with a GE TruSignal or Nellcor probe;  Philips Intellivue MP70 with a GE TruSignal Nellcor or Philips probe;  Welch Allyn with a Nellcor probe | Radiometer ABL800 | Arterial blood (no further detail) | Hospitalised adult patients (n = 400) | Mean 64.2 years | 72 to 100 | Skin pigmentation measured using the modified Fitzpatrick scale with categories of light, medium, or dark | Care setting, probe location, chronic respiratory failure-related health conditions, current tobacco smoking status, diabetes mellitus |
| Ries 1985 ^45^ | Hospital or wards | 2 models:  Ohmeda Biox IIA;  Hewlett-Packard 47201A oximeter | IL282 co-oximeter | Arterial blood (radial or other arteries) | Pulmonary patients who underwent clinical exercise testing (n = 136) | Adults, age not reported | Not reported | Skin pigmentation measured using a semi-quantitative scale of light to dark | Exercise loads, CoHgb, SaO_2_ ranges |
| Ries 1989 ^46^ | Hospital or wards | 2 models:  Ohmeda Biox III;  Hewlett-Packard 47201A oximeter | CO-oximeter | Arterial blood (radial or other arteries) | Pulmonary patients who underwent clinical exercise testing (n = 136) | Adults, age not reported | Not reported | Skin pigmentation measured using the Munsell colour system with categories of very light, light, average, and moderately dark or very dark |  |
| Ross 2014 ^47^ | Hospital or wards | 3 models:  Masimo pulse oximeters with Masimo LNCS probe, Nellcor pulse oximeters with Nellcor OxiMax probes, Masimo oximeters with Nellcor OxiMax probes | Radiometer ABL800 and Rapidlab 1265 (Siemens Healthcare), IL Gem 3000 | Arterial blood | ICU, mechanically ventilated children with cyanotic congenital heart disease (CCHD) or acute hypoxemic respiratory failure (n = 225) | Median 1 month for CCHD children, and 37 months for acute respiratory failure children | 72 to 96 | Race (African American, Hispanic, White, Asian, Other) | CCHD, prolonged capillary refill, having a SpO2 between 81% to 85%, 86% to 90%, or 91% to 95% (compared with a SpO2 of 96% to 97%), male gender, the combination of Masimo oximeter with a Nellcor sensor, mean airway pressure, hemoglobin, PICU site, temperature, fraction of inspired oxygen, age <2 months |
| Schallom 2018 ^48^ | Hospital or wards | 2 models:  Nellcor OxiMax Forehead sensor, Xhale Assurance nasal alar sensor | Radiometer ABL800 Flex Series blood gas analyser | Arterial blood | Critically ill adults (n = 43) | Mean 60.1 years | 70 to 98 | Ethnicity | NR |
| Smyth 1986 ^49^ | Lab | 2 models:  Hewlett-Packard oximeter, Ohmeda Biox II oximeter | Corning 175- blood gas analyser co-oximeter | Arterial blood (radial or other arteries) | Healthy Caucasian volunteers (n = 6) | Range: 23 to 33 year | Not reported | Race – all Caucasian | Oximeter model, oxygen saturation levels |
| Stewart 1991 ^50^ | Hospital or wards | 1 model:  Ohmeda Biox 3700 | Radiometer OSM2 | Arterial blood (radial or other arteries) | Adults with chronic rheumatic heart disease (n = 42) | Adults, age not reported | Not reported | Ethnicity - all Chinese | Presence of triscupid regurgitation, pulse oximeter location |
| Thrush 1994 ^51^ | Lab | 4 models:  Critikon Dinamap Plus Model 8700, Critikon Oxyshuttle, Ohmeda 3700, MiniOx IV | IL482 co-oximeter | Arterial blood (radial or other arteries) | Healthy, non-smoking adults (n = 22) | Mean 29 years | 80 to 100 | Race – all White | Hypoxemia severity |
| Valbuena 2021 (retrospective design) ^52^ | Hospital or wards | Not reported | Not reported, blood gas analysis | Arterial blood | Adult patients with respiratory failure or COVID-19 (n = 1562) | Not reported, > 18 years | Not reported | Ethnicity – White, Black, Hispanic, and Asian categories | Not reported |
| Vesoulis 2021 (retrospective design) ^53^ | Hospital or wards | 1 model:  Nellcor MAX-N adhesive SpO_2_ sensor (Covidien) (Philips IntelliVue MP70 or MX800 monitor) | Radiometer ABL800 Flex | Arterial blood | Preterm infants at neonatal intensive care unit (n = 294) | Median 4 days | Not reported | Ethnicity – White and Black categories | Not reported |
| Wiles 2021 (retrospective design) ^54^ | Hospital or wards | 1 model:  Nellcor probes (GE Healthcare B1x5 M/P monitor) | RAPIDpoint 500 analyser (Siemens Healthcare GmbH) | Arterial blood | Adult patients with COVID-19 pneumonitis (n = 194) | Mean 62 years | Not reported | Ethnicity – Asian, Black, White, and other categories | Not reported |
| Zeballos 1991 ^55^ | Lab | 2 models:  Hewlett-Packard 47201A;  Ohmeda Biox IIA | IL282 co-oximeter | Arterial blood (radial or other arteries) | Healthy, non-smoking volunteers (n = 33) | Mean 19 years | Not reported | Race – all dark pigmentation (Black volunteers) | Exercise levels, sea levels |
