# Supplementary material for: The accuracy of pulse oximetry in measuring oxygen saturation by levels of skin pigmentation: a systematic review and meta-analysis
Source: BMC Med. 2022 Aug 16;20:267. doi: 10.1186/s12916-022-02452-8 (PMC9377806; doi:10.1186/s12916-022-02452-8)
Supplement: Supplementary file 7 — Additional file 7: Table S3. Types of pulse oximeters and CO-oximetry evaluated in the included studies. [file 12916_2022_2452_MOESM7_ESM.docx]

## **Table S3. Types of pulse oximeters and CO-oximetry evaluated in the included studies**

| **Items** | **Summary statistics (n (%))** |
| --- | --- |
| Pulse oximeter manufacturers or brands (32 studies) |  |
| AFAC | 1 study (3.12%), including one model:   - AFAC FS10D [36] |
| AGPTEK | 1 study (3.12%), including one model:   - AGPTEK FS10C [36] |
| ANAPULSE | 1 study (3.12%), including one model:   - ANAPULSE ANP 100 [36] |
| CocoBear | 1 study (3.12%), including one model:   - Cocobear [36] |
| Contec | 1 study (3.12%), including one model:   - Contec CMS50D1 [36] |
| Criticare | 1 study (3.12%), including one model:   - Criticare CSI 501+ [30] |
| Critikon | 2 studies (6.25%), including two specified models:   - Oxyshuttle [51] - Dinamap Plus 8700 [51] - Unspecified model [40] |
| GE Healthcare | 1 study (3.12%), including two models:   - Carescape B450 monitor with Nellcor probe [43] - GE Dash 3000 [43] |
| Hewlett-Packard | 4 studies (12.50%), including one specified model:   - Hewlett-Packard 47201A [45; 46; 55] - Unspecified model [49] |
| HYLOGY | 1 study (3.12%), including one specified model:   - HYLOGY MD-H37 [36] |
| Masimo | 8 studies (25.00%), including at least five specified models:   - Radical 7 or Rainbow SET Radical 7 [31; 32; 43] - Masimo SET Quartz Q400 [43] - Masimo SET with LNCS sensor [34; 35]; - Masimo SET Blue sensor [34]; - Masimo SET Quartz (unspecified model) [43] - Unspecified model [28; 29; 47] |
| Mommed | 1 study (3.12%), including one specified model:   - Mommed YM101 [36] |
| MiniOx | 1 study (3.12%), including one model:   - MiniOx IV [51] |
| Minolta | 1 study (3.12%), including one model:   - Pulsox-7 [42] |
| Nellcor | 18 studies (56.25%), including ten specified models:   - D-25 [24] - Dura-Y [25] - DS-100A Durasensor sensor (SIEMENS SC1281 monitor) [37; 38] - MAX-N sensor (Philips IntelliVue MP70 or MX800 monitor) [53] - N-100 [23; 33] - N-200 [30] - N-595 [26; 31] - N-600 [34] - Oxiband [25] - OxiMax [32; 47; 48] - Unspecified model [28; 39; 40; 47; 54] |
| Nihon Koden | 1 study (3.12%), including one model:   - Nihon Koden [27] |
| Novametrix | 2 studies (6.25%), including two models:   - Novametrix 512 [43] - Novametrix 513s [26] |
| Nonin | 4 studies (12.50%), including seven specified models:   - Nonin 2120 [43] - Nonin 2140 [43] - Nonin Avant 4000 [43] - Nonin Avant 9700 [31; 43] - Nonin Lifesense Medair [43] - 3150 WristOx2 [35] - Onyx [26] - Nonin Avant (unspecified) [43] |
| Ohmeda | 11 studies (34.38%), including six specified models:   - Biox II [49] - Biox IIA [45; 55] - Biox III [46] - Biox 3700 [30; 39; 41; 50; 51] - Biox 3700E; [43] - 3740 [27] |
| Philips | 4 studies (12.50%), including two specified models:   - Philips M1191A (Philips IntelliVue MP70 monitor) [37] - Philips M1191BL [36] - Unspecified Philips oximeter (Philips IntelliVue MP70 monitor) [29; 43] |
| PRCMISEMED | 1 study (3.12%), including one model:   - PRCMISEMED F4PRO [26] |
| PULOX | 1 study (3.12%), including one model:   - PULOX PO-200 [26] |
| Simed | 2 studies (6.25%), including one specified model:   - S100e [27] - Unspecified model [40] |
| Welch Allyn | 1 study (3.12%), including one model:   - Welch Allyn [43] |
| Xhale Assurance | 1 study (3.12%), including one model:   - Xhale Assurance [48] |
| Zacurate | 1 study (3.12%), including one model:   - Zacurate Pro Series 500 DL [36] |
| Comparator co-oximeter devices (32 studies) |  |
| AVOXimeter | 1 study (3.12%), including one method:   - AVOXimeter 1000E co-oximeter [34] |
| Corning co-oximeter | 1 study (3.12%), including one method:   - Corning 175 blood gas analyser co-oximeter [49] |
| IL co-oximeter | 6 studies (18.75%), including three methods:   - IL 282 co-oximeter [45; 55] - IL 482 co-oximeter [27; 41; 51] - IL 682 co-oximeter [42] |
| Nova Stat Profile co-oximeter | 1 study (3.12%), including one method:   - Nova Stat Profile 3 pH/blood gas analyser [40] |
| Radiometer co-oximeter | 14 studies (43.75%), including six methods:   - Radiometer ABL520 [23] - Radiometer ABL625 [37; 38] - Radiometer ABL800 Flex co-oximeter [28; 29; 43; 48; 53] - Radiometer ABL90 Flex Plus [36] - Radiometer OSM2 [30; 33; 50] - Radiometer OSM3 [26; 31] |
| Siemens co-oximeter | 2 studies (6.25%), including two methods:   - Siemens Rapidlab 1265 analyser [32] - Siemens RAPIDpoint 500 analyser [54] |
| Combinations of different co-oximeters | 2 studies (6.25%), including two methods:   - 4-wavelength spectro-photometer, or co-oximeter (Radiometer OSM3) [24] - Radiometer ABL800 and Rapidlab 1265 (Siemens Healthcare), IL Gem 3000 [47] |
| Unspecified co-oximeter | 5 studies (15.62%), including:   - Unspecified methods [25; 35; 39; 46; 52] |

## 
