# Supplementary material for: The accuracy of pulse oximetry in measuring oxygen saturation by levels of skin pigmentation: a systematic review and meta-analysis
Source: BMC Med. 2022 Aug 16;20:267. doi: 10.1186/s12916-022-02452-8 (PMC9377806; doi:10.1186/s12916-022-02452-8)
Supplement: Supplementary file 8 — Additional file 8: Table S4. Mapping terms originally used for indicating skin pigmentation into low, medium or high level of skin pigmentation defined in the review for meta-analysis. [file 12916_2022_2452_MOESM8_ESM.docx]

## **Table S4. Mapping terms originally used for indicating skin pigmentation into low, medium or high level of skin pigmentation defined in the review for meta-analysis**

| **Skin pigmentation measurement methods** | **The number of classification categories as reported** | **Low (light) skin pigmentation** | **Medium skin pigmentation** | **High (dark) skin pigmentation** |
| --- | --- | --- | --- | --- |
| Fitzpatrick scale [29; 43; 44] | Three categories | ‘Light (Type I to Type II)’, or ‘light (score of 1 or 2)’ | ‘Medium (Type III to  Type IV)’, or ‘medium (score of 3 or 4)’ | ‘Dark (Type V to Type VI)’, or ‘dark (score of 5 or 6)’ |
| Munsell colour  system [24; 32] | Two categories [32] | ‘Light’ | - | ‘Dark’ |
|  | Three categories [24] | ‘Light’ | ‘Medium’ | ‘Dark’ |
| Using ethnicity to indicate skin pigmentation [26; 31; 55] | One category [55] | - | - | ‘Black’ participants |
|  | Two categories [26] | ‘Light (northern European)’ | - | ‘Dark (African-American)’ |
|  | Three categories [31] | ‘Light (Caucasian)’ | ‘Intermediate (Hispanic, Indian, Filipino, Vietnamese)’ | ‘Dark (African American)’ |
| Objective quantification using a reflectance spectrophotometer [27] | One category [27] |  |  | ‘Dark pigmentation’ |
