# Supplementary material for: The accuracy of pulse oximetry in measuring oxygen saturation by levels of skin pigmentation: a systematic review and meta-analysis
Source: BMC Med. 2022 Aug 16;20:267. doi: 10.1186/s12916-022-02452-8 (PMC9377806; doi:10.1186/s12916-022-02452-8)
Supplement: Supplementary file 9 — Additional file 9: Table S5. Evidence from studies where skin pigmentation measures cannot be specified or grouped into low, medium, and/or high pigmentation. [file 12916_2022_2452_MOESM9_ESM.docx]

## **Table S5. Evidence from studies where skin pigmentation measures cannot be specified or grouped into low, medium, and/or high pigmentation**

| **Measures of skin pigmentation** | **No. of participants (data pairs) and no. of studies and evaluations** | **Summary of reported results** | **Comments** |
| --- | --- | --- | --- |
| ***Subjective skin pigmentation categories related to ethnic groups (‘moderately pigmented’ or ‘racially pigmented’ skin, as reported, versus an unclear level of skin pigmentation)*** | 122 (267) in two studies with two evaluations [30, 33] | Skin pigmentation levels did not affect pulse oximetry accuracy:   - Across Ohmeda Biox 3700, Criticare CSI 501+, and Nellcor N200, the mean difference in bias between moderate pigmentation and others = 1.1% (SD 3), and skin pigmentation levels did not affect pulse oximetry accuracy.[30] - Nellcor N100 over-estimated oxygen saturation with a mean difference of 0.6%, and skin pigmentation levels did not affect pulse oximetry accuracy [33] | **Four models evaluated*:*** Ohmeda Biox 3700, Criticare CSI 501+, Nellcor N200,  Nellcor N100 |
| ***Massey score without specified categories*** | 74 (603) in two studies with five evaluations [34; 35] | Of the four models evaluated,   - There were no significant findings for the Masimo Standard sensor, Nellcor N600, and the WristOx2 3150 sensor regarding the effect of skin pigmentation on pulse oximetry accuracy.[34, 35] - Multivariable regression models for the mean bias from the Masimo Blue sensor yielded a significant effect for a Massey Score of 4 (p = 0.006), indicating an increase in average bias of 4% relative to individuals with a score of 1, adjusting for other demographic factors.[34] | **Four models evaluated:**  Masimo SET LNCS sensor (Masimo Standard); Masimo SET Blue sensor; Nellcor N-600 with Max-I sensor; Nonin (Bluetooth-enabled) WristOx2 3150 with 8008J sensors |
| ***An unnamed 4-level system with original categories of light to dark that were grouped into moderate and light pigmentation by the study authors*** | 23 (198) in one study with four evaluations [46] | Skin pigmentation was associated with the pulse oximetry accuracy for Hewlett-Packard ear oximeter 47201A but not for Biox IIA (Ohmeda)   - Differences between Hewlett-Packard oximeter readings and SaO_2_ were significantly larger in the five darker pigmented participants (16 data pairs) than in the 18 light pigmented ones (83 data pairs, t = -2.18, p < 0.05). It is unclear whether the larger difference suggested overestimation or underestimation. - SpO_2_ (Biox IIA)-SaO_2_ differences in darker pigmented participants were not significantly different from those in light pigmentation participants (t = 1.68, p > 0.05). | **Two models evaluated:**  Hewlett-Packard 47201A ear oximeter;  Ohmeda Biox IIA |
| ***Munsell system with four categories that could not be classified into low, medium, and high level of pigmentation*** | 154 (973) in one study with eight evaluations [46] | There was a higher overestimation of SpO_2_ in using Ohmeda Biox III in people with high pigmentation than those with low pigmentation. However, Hewlett-Packard 47201A oximeter did not overestimate SpO_2_ in either ‘very light’, ‘light’, ‘average’, or ‘moderately dark or very dark’ pigmentation groups of the Munsell system.   - Hewlett-Packard 47201A:   Very light – Arms = 3.23, mean bias (SD) = -0.30 (SD 3.22), limit of agreement = -6.61 to 6.01;  Light – Arms = 2.02, mean bias (SD) = 0 (2.02), limits of agreement = -3.96 to 3.96;  Average – Arms = 1.99, mean bias (SD) = -0.60 (1.90), limits of agreement = -4.32 to 3.12;  Moderately dark or very dark – Arms = 1.82, mean bias (SD) = -0.90 (1.58), limits of agreement = -4.00 to 2.20   - Ohmeda Biox III:   Very light – Arms = 2.40, mean bias (SD) = 0.60 (SD 2.32), limit of agreement = -3.95 to 5.15;  Light – Arms = 2.30, mean bias (SD) = 0.40 (2.27), limits of agreement = -4.04 to 4.84;  Average – Arms = 2.58, mean bias (SD) = 1.40 (2.17), limits of agreement = -2.85 to 5.65;  Moderately dark or very dark – Arms = 2.35, mean bias (SD) = 1.20 (2.02), limits of agreement = -2.76 to 5.16 | **Two models evaluated:**  Hewlett-Packard 47201A oximeter;  Ohmeda Biox III  This study did not reported numbers of participants for each level of skin pigmentation |
| ***Fitzpatrick scale with four categories that were classified by the authors to be two groups: IV-VI categories and I-III categories*** | 35 (2492) in one study with 11 evaluations [36] | SpO_2_-SaO_2_ bias, a continuous measure, was used for a multivariable logistic regression including Fitzpatrick scale and analyses showed that, compared with Fitzpatrick scale I-III categories, Fitzpatrick IV-VI categories as a group was significantly associated with bias for five models: AGPTEK FS10C regression beta 1.96 (SE 0.93), p = 0.04; Cocobear beta 2.30 (1.21), p = 0.05; HYLOGY MD-H37 beta 3.07 (1.13), p = 0.007; Mommed YM101 beta 2.40 (0.82), p = 0.004; and Zacurate Pro Series 500DL beta 2.34 (1.1), p = 0.038. All beta values are positive and larger than 1, suggesting a higher skin pigmentation means a higher bias. However, the association was not significant for the other pulse oximetry models: AFAC FS10D, ANAPULSE ANP 100, Contec CMS50D1, PRCMISEMED F4 PRO, and PULOX-PO-200, all with p > 0.05 |  |
