# Supplementary material for: The accuracy of pulse oximetry in measuring oxygen saturation by levels of skin pigmentation: a systematic review and meta-analysis
Source: BMC Med. 2022 Aug 16;20:267. doi: 10.1186/s12916-022-02452-8 (PMC9377806; doi:10.1186/s12916-022-02452-8)
Supplement: Supplementary file 10 — Additional file 10: Figure S1. Summary presentations of study sample sizes (n) and numbers of data pairs compared (N), accuracy root mean square (Arms), mean bias (SD) and limits of agreement (LoA) of pulse oximeters for the subgroup of high (dark) skin pigmentation. [file 12916_2022_2452_MOESM10_ESM.docx]

## **Figure S1. Summary presentations of study sample sizes (n) and numbers of data pairs compared (N), accuracy root mean square (Arms), mean bias (SD) and limits of agreement (LoA) of pulse oximeters for the subgroup of high (dark) skin pigmentation**


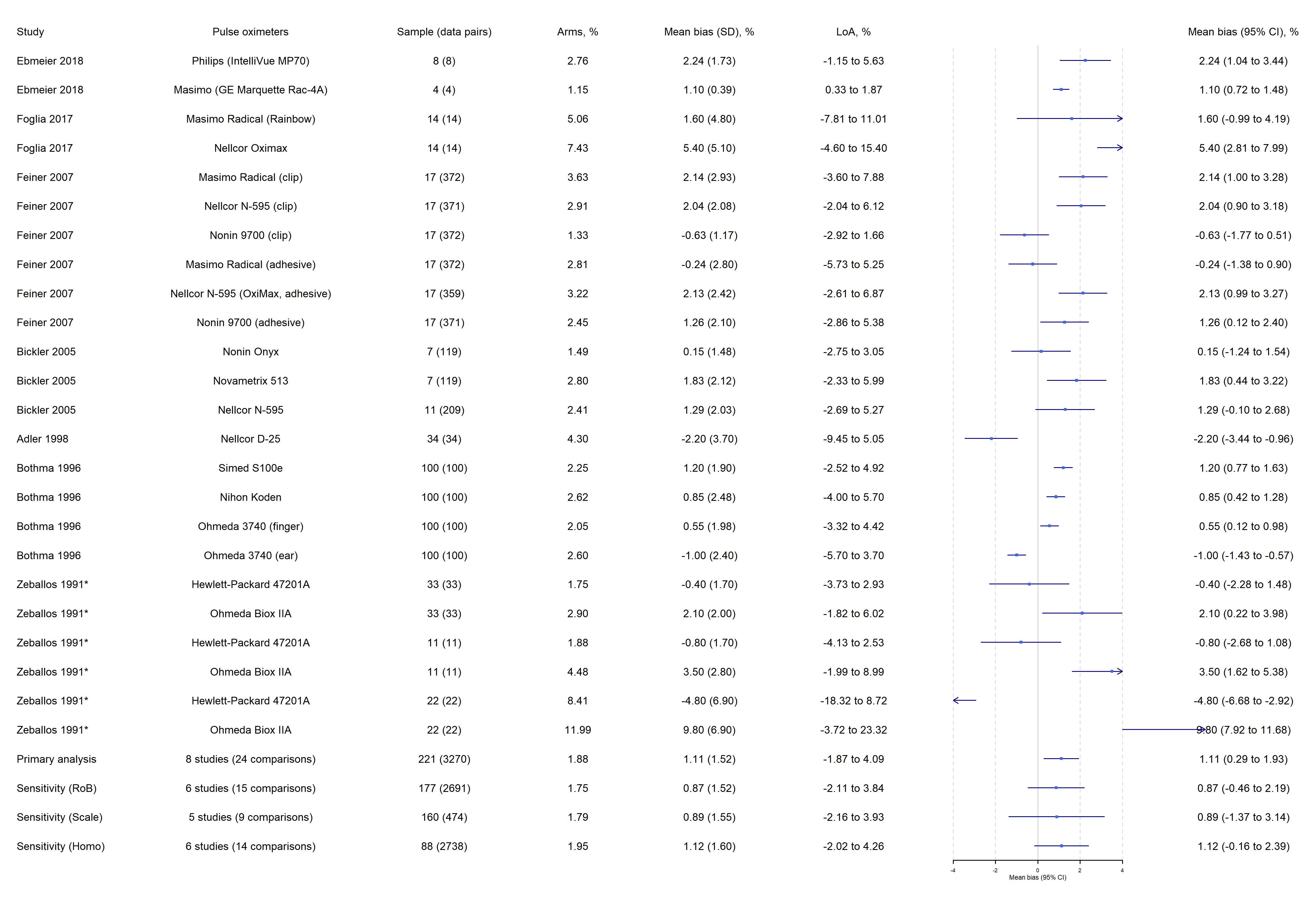


Note:

- The Chi^2^ test for heterogeneity in the primary analysis suggested a Q(df = 23) = 2251.16, with P value < 0.0001.
- Tau^2^ between the 8 studies = 0 (95% CI 0 to 3.77); Tau^2^ between the 24 comparisons = 7.16 (4.14 to 13.81).
- The estimated overall I^2^ for the primary analysis = 98.03%, of which about 0% is due to between-studies heterogeneity, and 98.03% due to within-study heterogeneity.
