# Supplementary material for: The accuracy of pulse oximetry in measuring oxygen saturation by levels of skin pigmentation: a systematic review and meta-analysis
Source: BMC Med. 2022 Aug 16;20:267. doi: 10.1186/s12916-022-02452-8 (PMC9377806; doi:10.1186/s12916-022-02452-8)
Supplement: Supplementary file 11 — Additional file 11: Figure S2. Summary presentations of study sample sizes (n) and numbers of data pairs compared (N), accuracy root mean square (Arms), mean bias (SD) and limits of agreement (LoA) of pulse oximeters for the subgroup of medium skin pigmentation. [file 12916_2022_2452_MOESM11_ESM.docx]

## **Figure S2. Summary presentations of study sample sizes (n) and numbers of data pairs compared (N), accuracy root mean square (Arms), mean bias (SD) and limits of agreement (LoA) of pulse oximeters for the subgroup of medium skin pigmentation**


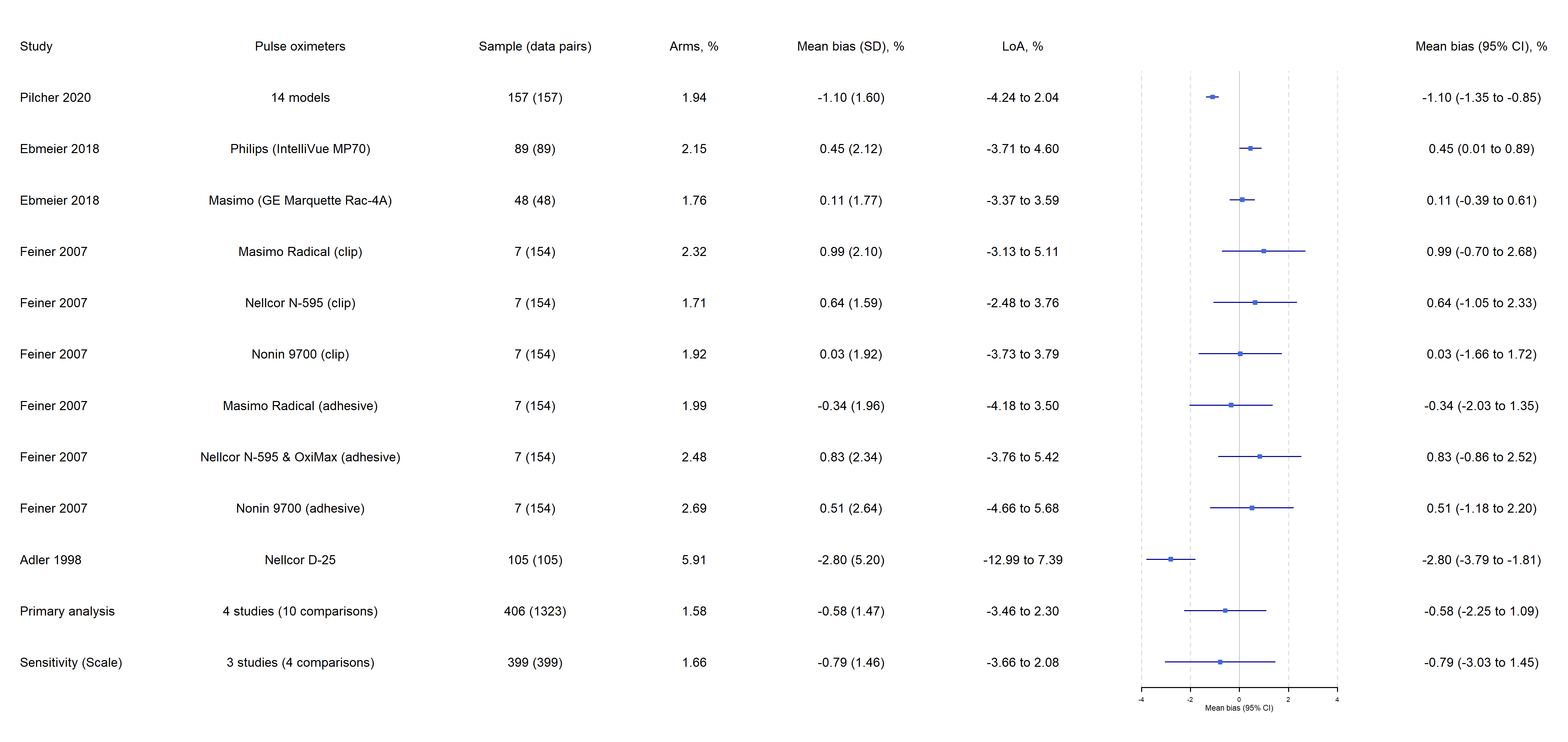


Note:

- The Chi^2^ test for heterogeneity in the primary analysis suggested a Q(df = 9) = 81.92, with P value < 0.0001.
- Tau^2^ between the 4 studies = 1.47 (95% CI 0 to 10.91); Tau^2^ between the 10 comparisons = 0.18 (0.02 to 1.17).
- The estimated overall I^2^ for the primary analysis = 92.65%, of which about 82.39% is due to between-studies heterogeneity, and 10.25% due to within-study heterogeneity.
