# Supplementary material for: The accuracy of pulse oximetry in measuring oxygen saturation by levels of skin pigmentation: a systematic review and meta-analysis
Source: BMC Med. 2022 Aug 16;20:267. doi: 10.1186/s12916-022-02452-8 (PMC9377806; doi:10.1186/s12916-022-02452-8)
Supplement: Supplementary file 12 — Additional file 12: Figure S3. Summary presentations of study sample sizes (n) and numbers of data pairs compared (N), accuracy root mean square (Arms), mean bias (SD) and limits of agreement (LoA) of pulse oximeters for the subgroup of low (light) skin pigmentation. [file 12916_2022_2452_MOESM12_ESM.docx]

## **Figure S3. Summary presentations of study sample sizes (n) and numbers of data pairs compared (N), accuracy root mean square (Arms), mean bias (SD) and limits of agreement (LoA) of pulse oximeters for the subgroup of low (light) skin pigmentation**


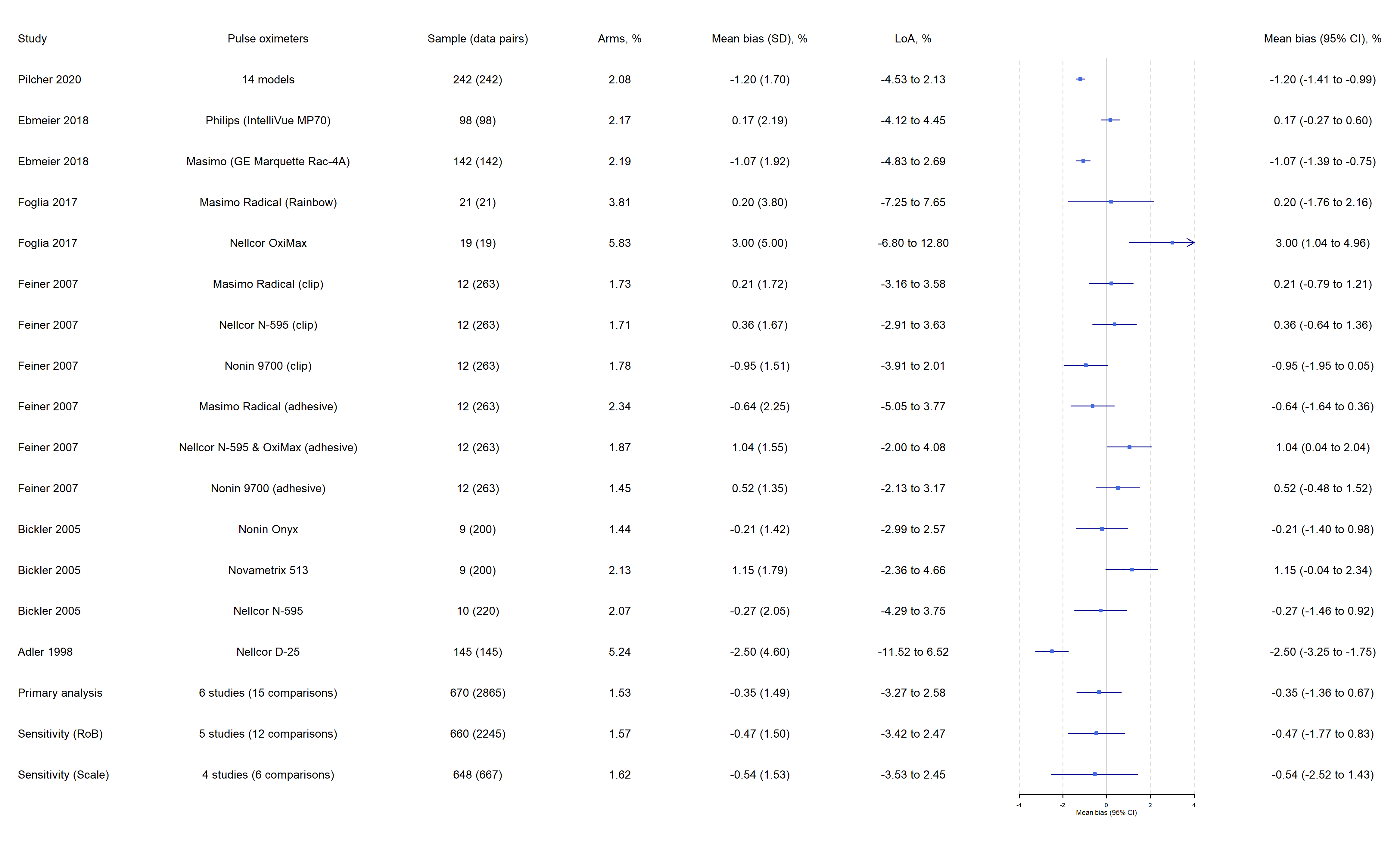


Note:

- The Chi^2^ test for heterogeneity in the primary analysis suggested a Q(df = 14) = 243.72, with P value < 0.0001.
- Tau^2^ between the 6 studies = 0.32 (95% CI 0 to 4.87); Tau^2^ between the 15 comparisons = 0.99 (0.37 to 2.96).
- The estimated overall I^2^ for the primary analysis = 92.73%, of which about 22.42% is due to between-studies heterogeneity, and 70.31% due to within-study heterogeneity.
