# Supplementary material for: The accuracy of pulse oximetry in measuring oxygen saturation by levels of skin pigmentation: a systematic review and meta-analysis
Source: BMC Med. 2022 Aug 16;20:267. doi: 10.1186/s12916-022-02452-8 (PMC9377806; doi:10.1186/s12916-022-02452-8)
Supplement: Supplementary file 13 — Additional file 13: Table S6. Summary of findings table for the impact of skin pigmentation and ethnicity on the accuracy of pulse oximetry compared with CO-oximetry. [file 12916_2022_2452_MOESM13_ESM.docx]

## **Table S6. Summary of findings table for the impact of skin pigmentation and ethnicity on the accuracy of pulse oximetry compared with CO-oximetry**

| Outcomes | **Anticipated absolute effects^*^** (95% CI) | | Mean bias (mean SpO_2_-SaO_2_) (95% CI) | № of participants (studies) | Certainty of the evidence (GRADE) | Comments |
| --- | --- | --- | --- | --- | --- | --- |
|  | **Oxygen saturation measured by pulse oximetry (SpO_2_)** | **Actual oxygen saturation measured by CO-oximetry (SaO_2_)** |  |  |  |  |
| Mean bias in people with high (dark) skin pigmentation | 90% | **89%** (90% to 88%) | **Mean bias 1.11** (0.29 to 1.93) | 221 participants with 3270 SpO_2_-SaO_2_ pairs (8 studies with 24 comparison evaluations) | ⨁⨁⨁⊝ Moderate ^a^ | Pulse oximetry SpO_2_ readings probably overestimate arterial oxygen saturation by on average 1.11% compared with the SaO_2_ measure of CO-oximetry in people with high (dark) skin pigmentation. |
| Mean bias in people with medium skin pigmentation | 90% | **90%** (89% to 92%) | **Mean bias -0.58** (-2.25 to 1.09) | 406 participants with 1323 SpO_2_-SaO_2_ pairs (4 studies with 10 comparison evaluations) | ⨁⊝⊝⊝ Very low ^a,b^ | It is uncertain if pulse oximetry would overestimate arterial oxygen saturation compared with the use of CO-oximetry in people with medium skin pigmentation. |
| Mean bias in people with low (light) skin pigmentation | 90% | **90**% (89% to 91%) | **Mean bias -0.35** (-1.36 to 0.67) | 670 participants with 2865 SpO_2_-SaO_2_ pairs (6 studies with 15 comparison evaluations) | ⨁⨁⊝⊝ Low^a,c^ | Pulse oximetry may not overestimate arterial oxygen saturation compared with the use of CO-oximetry in people with low (light) skin pigmentation. |
| Mean bias in people from Black/African American ethnic groups | 90% | **89%** (89% to 88%) | **Mean bias 1.52** (0.95 to 2.09) | 459 participants with 5753 SpO_2_-SaO_2_ pairs (9 studies with 22 comparison evaluations) | ⨁⨁⊝⊝ Low ^d,e^ | Pulse oximetry SpO_2_ readings may overestimate arterial oxygen saturation by on average 1.52% compared with the SaO_2_ measure of CO-oximetry in people from Black/African American ethnic groups. |
| Mean bias in people of ethnicity other than Black or White such as Asians, Hispanics, those of mixed ethnicity | 90% | **90%** (90% to 89%) | **Mean bias 0.31** (0.09 to 0.54) | 522 participants with 2646 SpO_2_-SaO_2_ pairs (3 studies with 9 comparison evaluations) | ⨁⨁⊝⊝ Low ^d,e^ | Pulse oximetry may very slightly overestimate arterial oxygen saturation compared with the use of CO-oximetry in people of ethnicity other than Black and White such as Asians, Hispanics, those of mixed ethnicity. |
| Mean bias in people from White/Caucasian ethnic groups | 90% | **89%** (89% to 90%) | **Mean bias 0.55** (-0.21 to 1.31) | 2195 participants with 12870 SpO_2_-SaO_2_ pairs (13 studies with 48 comparison evaluations) | ⨁⊝⊝⊝ Very low^a,e,f^ | It is uncertain if pulse oximetry would overestimate or underestimate arterial oxygen saturation compared with the use of CO-oximetry in White/Caucasians. |
| ***The actual oxygen saturation measured by CO-oximetry (SaO_2_)** (and its 95% confidence interval) is based on the assumed oxygen saturation measured by pulse oximetry (SpO_2_) of 90% and the **relative effect** (and its 95% CI). | | | | | | |
| **GRADE Working Group grades of evidence** **High certainty:** we are very confident that the true effect lies close to that of the estimate of the effect. **Moderate certainty:** we are moderately confident in the effect estimate: the true effect is likely to be close to the estimate of the effect, but there is a possibility that it is substantially different. **Low certainty:** our confidence in the effect estimate is limited: the true effect may be substantially different from the estimate of the effect. **Very low certainty:** we have very little confidence in the effect estimate: the true effect is likely to be substantially different from the estimate of effect. | | | | | | |

#### Explanations

a. Downgraded once for the joint consideration of inconsistency and publication bias. Firstly, the analysis found either high statistical heterogeneity, differences between studies in pulse oximetry devices, and/or the large variation of point estimates on the forest plot. Secondly, despite a comprehensive search, only part of the included studies presented data for meta-analysis and only English-language publications were searched for.

b. Downgraded twice for imprecision. The limits of the CI are very large and cover values that lead to different conclusions on pulse oximetry’s accuracy: e.g., the lower limit suggests a clear underestimation whilst the upper limit suggests a clear overestimation.

c. Downgraded once for imprecision. The limits of the CI are slightly wide and the range covers values that potentially lead to different conclusions on pulse oximetry’s accuracy: e.g., the lower limit suggests a small underestimation. The upper limit suggests a small overestimation.

d. Downgraded once for the joint consideration of study limitations and publication bias. Firstly, a proportion of the included studies and data were at high overall risk of bias. Secondly, despite a comprehensive search, only part of the included studies presented data for meta-analysis and only English-language publications were searched for.

e. Downgraded once for the indirectness. The evidence from data synthesis for ethnic groups was indirectly relevant to the topic of skin pigmentation for this review.

f. Downgraded once for study limitations. In the meta-analysis, around half of the included studies and/or data were at high overall risk of bias.
