# Supplementary material for: The accuracy of pulse oximetry in measuring oxygen saturation by levels of skin pigmentation: a systematic review and meta-analysis
Source: BMC Med. 2022 Aug 16;20:267. doi: 10.1186/s12916-022-02452-8 (PMC9377806; doi:10.1186/s12916-022-02452-8)
Supplement: Supplementary file 14 — Additional file 14: Figure S4. Summary presentations of study sample sizes (n) and numbers of data pairs compared (N), accuracy root mean square (Arms), mean bias (SD) and limits of agreement (LoA) of pulse oximeters for levels of skin pigmentation by the different types of pulse oximeters. [file 12916_2022_2452_MOESM14_ESM.docx]

## **Figure S4. Summary presentations of study sample sizes (n) and numbers of data pairs compared (N), accuracy root mean square (Arms), mean bias (SD) and limits of agreement (LoA) of pulse oximeters for levels of skin pigmentation by the different types of pulse oximeters**


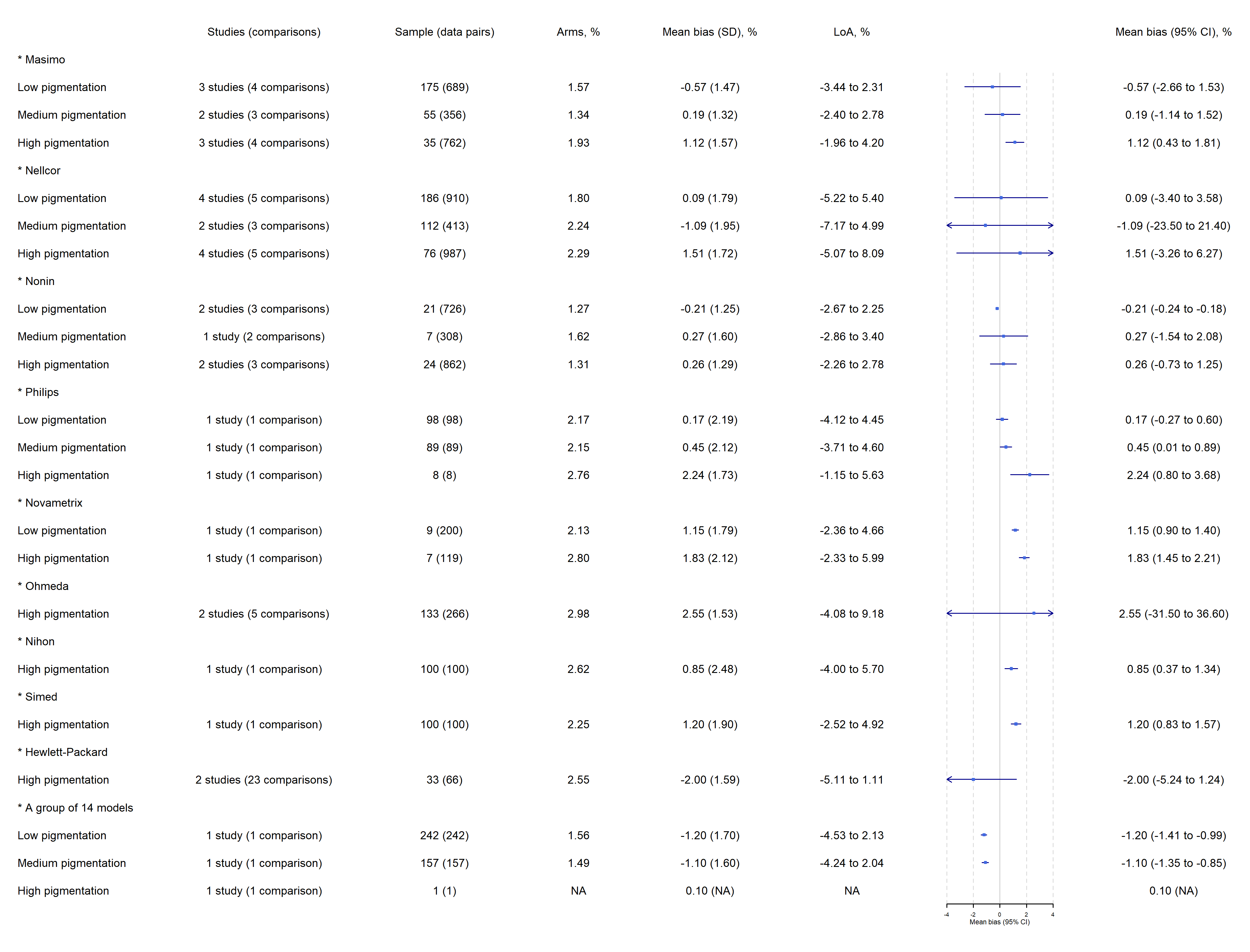


This figure presents the impact of skin pigmentation on pulse oximetry accuracy according to types of pulse oximeters evaluated. Results of analyses suggested that:

- Masimo, Nellcor, Philips, Novametrix, Ohmeda, Nihon, and Simed appear to have higher SpO_2_ readings than SaO_2_ by on average 1% in people with high skin pigmentation.
- Hewlett-Packard appears to produce SpO_2_ measures 2% lower than SaO_2_ readings in people with high skin pigmentation.
- Novametrix produces a SpO_2_ measure higher than SaO_2_ readings by on average 1% in people with low (light) skin pigmentation whilst others of these devices appear to produce SpO_2_ measures with a bias no more than 1% compared with SaO_2_ readings in people from medium and low skin pigmentation subgroups.
- Nonin does not result in over- or underestimation of oxygen saturation in people with any level of skin pigmentation.
