# Supplementary material for: The accuracy of pulse oximetry in measuring oxygen saturation by levels of skin pigmentation: a systematic review and meta-analysis
Source: BMC Med. 2022 Aug 16;20:267. doi: 10.1186/s12916-022-02452-8 (PMC9377806; doi:10.1186/s12916-022-02452-8)
Supplement: Supplementary file 15 — Additional file 15: Table S7. Evidence from studies that could not be included in quantitative data pooling for the ethnicity factor. [file 12916_2022_2452_MOESM15_ESM.docx]

## **Table S7. Evidence from studies that could not be included in quantitative data pooling for the ethnicity factor**

| **Measures of skin pigmentation** | **No. of participants (data pairs) and no. of studies and evaluations** | **Summary of reported results** | **Comments** |
| --- | --- | --- | --- |
| ***Ethnic groups: ‘moderately pigmented’ or ‘racially pigmented’ as reported, versus unclear*** | 122 (267) in two studies with two evaluations [30, 33] | ‘Racially pigmented’ skin did not affect pulse oximetry accuracy:   - Across Ohmeda Biox 3700, Criticare CSI 501+, and Nellcor N200, the mean difference in bias between moderate pigmentation and others = 1.1% (SD 3), and skin pigmentation levels did not affect pulse oximetry accuracy.[30] - Nellcor N100 over-estimated oxygen saturation with a mean difference of 0.6%, and skin pigmentation levels did not affect pulse oximetry accuracy [33] | **Four models evaluated*:*** Ohmeda Biox 3700, Criticare CSI 501+, Nellcor N200,  Nellcor N100 |
| ***Ethnic groups: Aboriginal and/or Torres Strait Islander [ATSI] vs non-ATSI*** | 929 (18650) in one study with one evaluation [28] | Based on categories of mean bias > 3% vs <= 3% as the outcome measure, an univariate analysis produced OR of 0.94 (95% 0.60 to 1.48), p = 0·790, meaning ATSI was not associated with a bias higher than 3% compared with non-ATSI. Multivariate analysis produced a OR of 1.29 (95% CI 0.99 to 1.68), p = 0·055, meaning ATSI was marginally associated with a bias higher than 3% compared with non-ATSI. | **Two models evaluated together:**  Masimo, Nellcor (Covidien) |
| ***Race/ethnic groups: Chinese, Malay, and Indian*** | 33 (150 readings noted, but only 98 presented) in one study with three evaluations [40] | There was a significant difference between the groups (ANOVA, p<0.05) with the Indian group having the greatest difference between SpO_2_ and SaO_2_, Malay having the moderate difference, and Chinese having the least difference. | **Three models evaluated**  Nellcor, Simed, Critikon |
| ***Race/ethnic groups: African American, Hispanic, White, Asian, and Other groups*** | 225 (1980) in one study with one evaluation [47] | Based on binary categories of mean bias of < 3% and >3%, a multivariable analysis produced regression coefficients = African American -0.55 (p= .003), Hispanic -0.15 (NS), Asian -0.26 (NS), and Other -0.03 (NS), all compared with White group as the reference. A secondary analysis suggested 'a lower likelihood of bias was associated with African American race/ethnicity’. The results suggested a lower bias in African American ethnic groups than the White group. | **Three models evaluated**  Masimo LCNS pulse oximeters, Nellcor oximeters (OxiMax probes), Masimo oximeters (OxiMax disposable probes) |
| ***Race/ethnic groups: Caucasian vs Black/African American*** | 43 (136) in one study with two evaluations[48] | - Nellcor OxiMax Forehead sensor   Chi-square OR (within 3% of SaO2 as the cut off): Caucasians were 1.2 times more likely to have a clinically accurate forehead measurement than African Americans. However, this association was not statistically significant (p = 0.74).   - Xhale Assurance nsal alar sensor   Chi-square OR (within 3% of SaO2 as the cut off): Caucasians were 2.65 times more likely to have a clinically accurate nasal measurement than African Americans. This association was statistically significant (p = 0.04) | **Two models evaluated**  Nellcor OxiMax Forehead sensor, Xhale Assurance nsal alar sensor |
| ***Ethnic group: all Caucasian*** | 6 (NR) in one study with two evaluations [49] | The author fitted a regression line between SpO_2_ and SaO_2_. No data on mean bias was reported | **Two models evaluated**  Hewlett Packard (HP) oximeter and Biox II oximeter |
| ***Ethnic group: all Chinese*** | 42 (NR) in one study with one evaluation [50] | The authors reported median bias of using the Ohmeda Biox 3700 model in (1) adult patients who were scheduled to undergo open heart surgery and (2) those who did not have tricuspid regurgitation by probe sites (finger and ear).   - Ear, patients who were scheduled to undergo open heart surgery: median (range) = 4% (0 to + 11 %), - Ear, patients who did not have tricuspid regurgitation: median (range) = 1% (0 to +4%), - Finger, patients who were scheduled to undergo open heart surgery: median (range) = 3% (-2 to + 10%), and - Finger, patients who did not have tricuspid regurgitation: median (range) = 1% (- 1 to + 5%). | **One model evaluated**  Ohmeda Biox 3700 |
