# Supplementary material for: The accuracy of pulse oximetry in measuring oxygen saturation by levels of skin pigmentation: a systematic review and meta-analysis
Source: BMC Med. 2022 Aug 16;20:267. doi: 10.1186/s12916-022-02452-8 (PMC9377806; doi:10.1186/s12916-022-02452-8)
Supplement: Supplementary file 17 — Additional file 17: Figure S6. Summary presentations of study sample sizes (n) and numbers of data pairs compared (N), accuracy root mean square (Arms), mean bias (SD) and limits of agreement (LoA) of pulse oximeters for the subgroup of non-Black, non-White ethnic groups. [file 12916_2022_2452_MOESM17_ESM.docx]

## **Figure S6. Summary presentations of study sample sizes (n) and numbers of data pairs compared (N), accuracy root mean square (Arms), mean bias (SD) and limits of agreement (LoA) of pulse oximeters for the subgroup of non-Black, non-White ethnic groups**


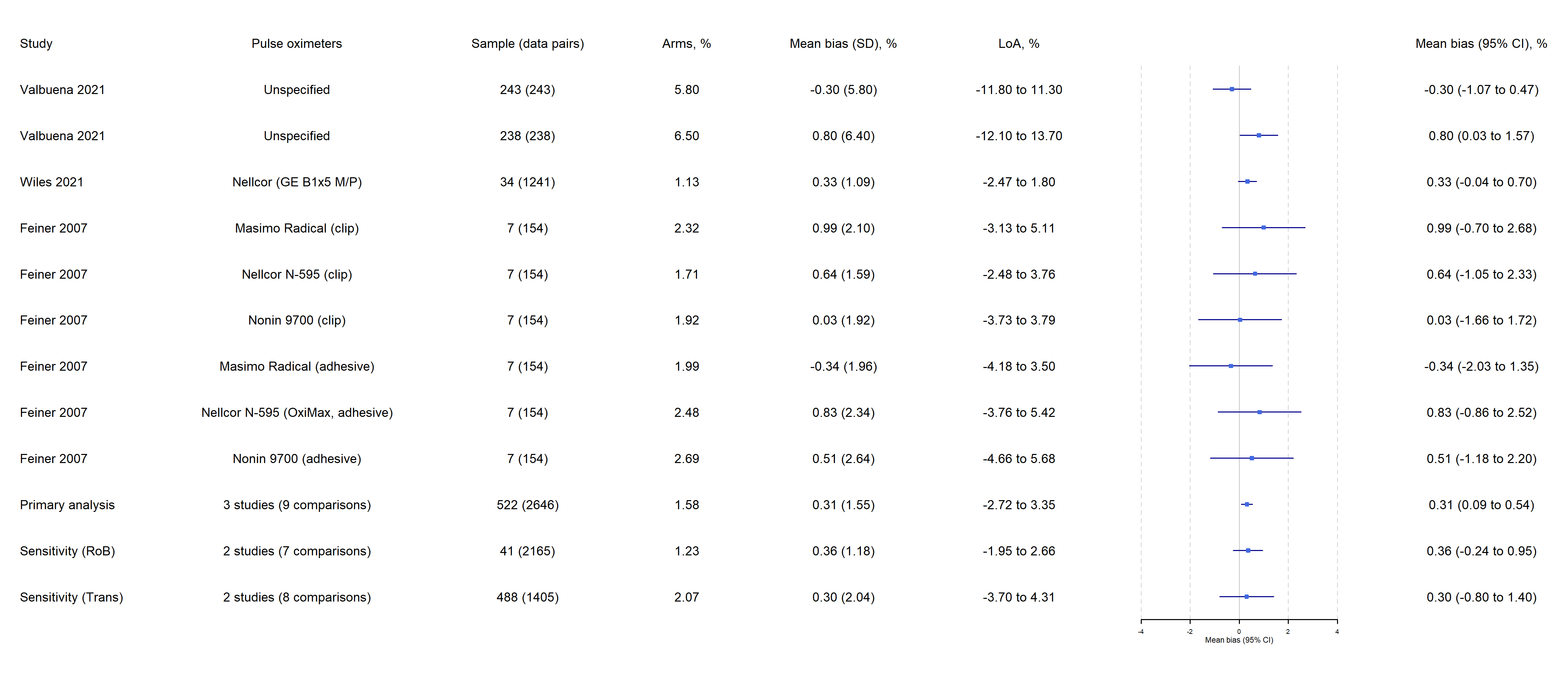


Note:

- The Chi^2^ test for heterogeneity in the primary analysis suggested a Q(df = 8) = 56.20, with P value < 0.0001.
- Tau^2^ between the 3 studies = 0 (95% CI 0 to 2.34); Tau^2^ between the 9 comparisons = 0.23 (0.07 to 0.90).
- The estimated overall I^2^ for the primary analysis = 47.95%, of which about 0% is due to between-studies heterogeneity, and 47.95% due to within-study heterogeneity.
