# Supplementary material for: The accuracy of pulse oximetry in measuring oxygen saturation by levels of skin pigmentation: a systematic review and meta-analysis
Source: BMC Med. 2022 Aug 16;20:267. doi: 10.1186/s12916-022-02452-8 (PMC9377806; doi:10.1186/s12916-022-02452-8)
Supplement: Supplementary file 18 — Additional file 18: Figure S7. Summary presentations of study sample sizes (n) and numbers of data pairs compared (N), accuracy root mean square (Arms), mean bias (SD) and limits of agreement (LoA) of pulse oximeters for the subgroup of White/Caucasian ethnic groups. [file 12916_2022_2452_MOESM18_ESM.docx]

## **Figure S7. Summary presentations of study sample sizes (n) and numbers of data pairs compared (N), accuracy root mean square (Arms), mean bias (SD) and limits of agreement (LoA) of pulse oximeters for the subgroup of White/Caucasian ethnic groups**


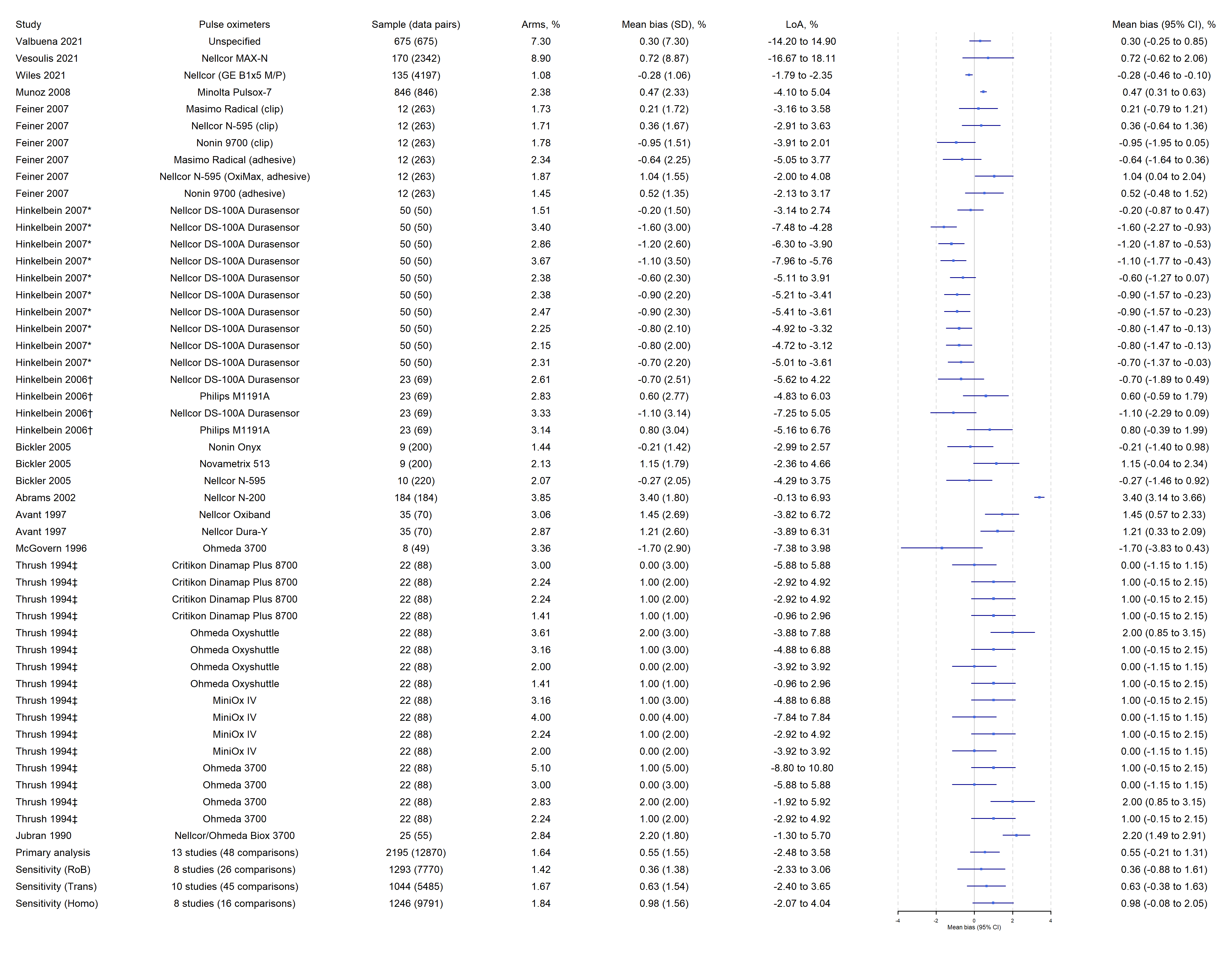


Note:

- The Chi^2^ test for heterogeneity in the primary analysis suggested a Q(df = 47) = 1100.39, with P value < 0.0001.
- Tau^2^ between the 13 studies = 1.10 (95% CI 0.31 to 3.48); Tau^2^ between the 9 comparisons = 0.38 (0.24 to 0.67).
- The estimated overall I^2^ for the primary analysis = 94.39%, of which about 69.92% is due to between-studies heterogeneity, and 24.47% due to within-study heterogeneity.
