# Supplementary material for: The accuracy of pulse oximetry in measuring oxygen saturation by levels of skin pigmentation: a systematic review and meta-analysis
Source: BMC Med. 2022 Aug 16;20:267. doi: 10.1186/s12916-022-02452-8 (PMC9377806; doi:10.1186/s12916-022-02452-8)
Supplement: Supplementary file 19 — Additional file 19: Figure S8. Summary presentations of study sample sizes (n) and numbers of data pairs compared (N), accuracy root mean square (Arms), mean bias (SD) and limits of agreement (LoA) of pulse oximeters for ethnic groups by the different types of pulse oximeters. [file 12916_2022_2452_MOESM19_ESM.docx]

## **Figure S8. Summary presentations of study sample sizes (n) and numbers of data pairs compared (N), accuracy root mean square (Arms), mean bias (SD) and limits of agreement (LoA) of pulse oximeters for ethnic groups by the different types of pulse oximeters**


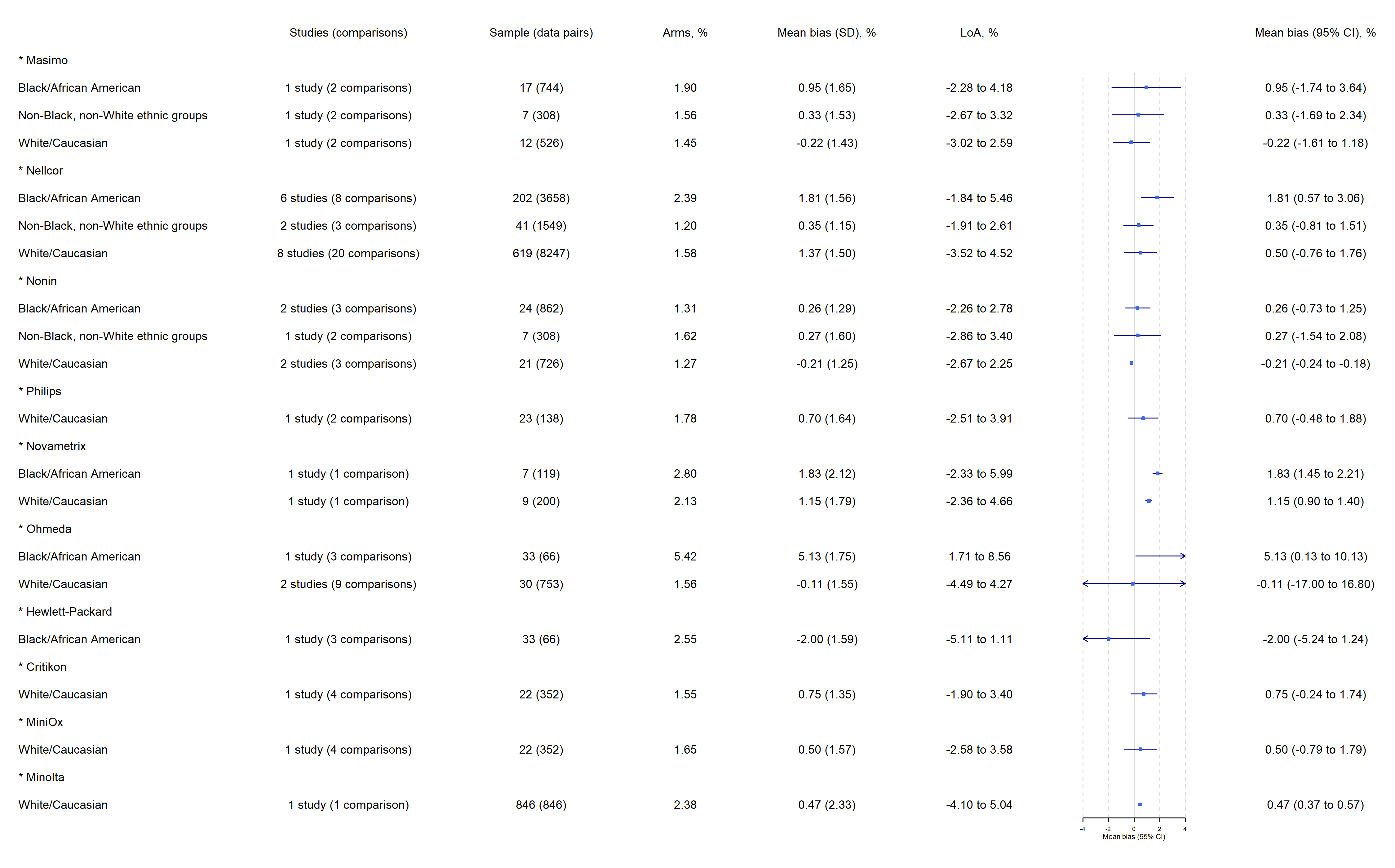


This figure presents the impact of ethnicity on pulse oximetry accuracy according to types of pulse oximeters evaluated. Results of analyses suggested that:

- Masimo, Nellcor, Novametrix, and Ohmeda appear to have higher SpO_2_ measures than SaO_2_ readings by on average 1% in people from Black/African American ethnic groups.
- Hewlett-Packard appears to obtain SpO_2_ by 2% lower than SaO_2_ readings in people with high skin pigmentation.
- Novametrix gives a SpO_2_ measure higher than SaO_2_ readings by on average 1% in people from White/Caucasian ethnic groups whilst others of these devices appear to produce SpO_2_ measures with a bias no more than 1% compared with SaO_2_ readings in White/Caucasians and those from ethnicity other than Black or White.
- Nonin does not result in over- or underestimation of oxygen saturation in people from any ethnic group.
